# Implementation and User Evaluation of an eHealth Technology Platform Supporting Patients With Cardiovascular Disease in Managing Their Health After a Cardiac Event: Mixed Methods Study

**DOI:** 10.2196/43781

**Published:** 2023-03-24

**Authors:** Britt E Bente, Jobke Wentzel, Celina Schepers, Linda D Breeman, Veronica R Janssen, Marcel E Pieterse, Andrea W M Evers, Lisette van Gemert-Pijnen

**Affiliations:** 1 Department of Psychology, Health, and Technology Faculty of Behavioral, Management and Social Sciences University of Twente Enschede Netherlands; 2 Department of Health Care and Social Work University of Applied Sciences Windesheim Zwolle Netherlands; 3 Unit of Health, Medical, and Neuropsychology Faculty of Social and Behavioral Sciences Leiden University Leiden Netherlands; 4 Department of Cardiology Leiden University Medical Center Leiden Netherlands

**Keywords:** patient needs, health behavior, lifestyle support, user-centered design, implementation, evaluation, cardiovascular disease, app, web-based platform, intervention

## Abstract

**Background:**

eHealth technology can help patients with cardiovascular disease adopt and maintain a healthy lifestyle by supporting self-management and offering guidance, coaching, and tailored information. However, to support patients over time, eHealth needs to blend in with their needs, treatment, and daily lives. Just as needs can differ between patients, needs can change within patients over time. To better adapt technology features to patients’ needs, it is necessary to account for these changes in needs and contexts of use.

**Objective:**

This study aimed to identify and monitor patients’ needs for support from a web-based health management platform and how these needs change over time. It aimed to answer the following research questions: “How do novice and more advanced users experience an online health management platform?” “What user expectations support or hinder the adoption of an online health management platform, from a user perspective?” and “How does actual usage relate to user experiences and adoption?”

**Methods:**

A mixed methods design was adopted. The first method involved 2 rounds of usability testing, followed by interviews, with 10 patients at 0 months (round 1) and 12 patients at 6 months (round 2). In the second method, log data were collected to describe the actual platform use.

**Results:**

After starting cardiac rehabilitation, the platform was used frequently. The patients mentioned that they need to have an incentive, set goals, self-monitor their health data, and feel empowered by the platform. However, soon after the rehabilitation program stopped, use of the platform declined or patients even quit because of the lack of continued tailored or personalized advice. The reward system motivated them to log data, but most participants indicated that being healthy should be the main focus, not receiving gifts. A web-based platform is flexible, accessible, and does not have any obligations; however, it should be implemented as an addition to regular care.

**Conclusions:**

Although use of the platform declined in the longer term, patients quitting the technology did not directly indicate that the technology was not functioning well or that patients no longer focused on achieving their values. The key to success should not be user adherence to a platform but adherence to healthy lifestyle habits. Therefore, the implementation of eHealth should include the transition to a stage where patients might no longer need support from a technology platform to be independently and sustainably adherent to their healthy lifestyle habits. This emphasizes the importance of conducting multi-iterative evaluations to continuously monitor whether and how patients’ needs and contexts of use change over time. Future research should focus on how this transition can be identified and monitored and how these insights can inform the design and implementation of the technology.

## Introduction

### Background

Supporting patients with cardiovascular disease (CVD) in adopting and maintaining a healthy lifestyle is a challenging and ongoing process. A healthy lifestyle is often not limited to one action or change but requires ongoing attention. eHealth technology can help patients with CVD in tackling this challenge by supporting self-management and offering guidance, coaching, and information. eHealth enables patients to access their health data [1] and receive feedback on their behavior and health [2] and provides tips and support to improve their health. These insights and feedback increase the self-management ability of patients [1], which is necessary to adopt and maintain a healthy lifestyle even at home when cardiac rehabilitation has ended. In addition, the possibility of sharing self-monitored data with health care professionals provides more insights into patients’ health than would be possible during a consultation [3], which could result in more personalized treatment choices. However, achieving long-lasting effects of eHealth is possible only if patients (or users) feel engaged, and even if they do, it does not mean that they are adherent [4,5]. To improve patients’ engagement and adherence and thus be able to assist them over time, the eHealth technology needs to blend in with their treatment and daily lives [6-8]. This emphasizes the importance of intertwining implementation (to identify and tackle potential challenges) within the development of eHealth [9].

In this study, we focus on the user-centered development and implementation of a particular web-based lifestyle platform: the Vital10 Personal Health Platform (Vital10 PHP). This platform aims to support patients with CVD with adopting and maintaining a healthy lifestyle in their own home situation [10]. The theoretical framework behind the user-centered design approach adopted in this study is the CeHRes Roadmap, a holistic and participatory approach for eHealth development, implementation, and evaluation [9]. This road map emphasizes the importance of stakeholder involvement (eg, patients as users), which includes the identification of their values and needs and translation of these values to specific requirements for the design of the eHealth technology. A key principle of the CeHRes Roadmap is that user-centered design involves a continuous, multi-iterative process of user evaluation rather than a one-size-fits-all approach. In a previous study, we identified the values of patients with CVD for support from a web-based health management platform [6]. These values (ranging from the need for security, support, and reduction in anxiety to the need for the tailoring of treatment and personalized and accessible care) informed the development of the Vital10 PHP. Although all the (design) features of the Vital10 PHP are based on these identified values, continued monitoring among users during implementation is essential to assess the extent to which the platform satisfies these values [9]. Moreover, just as needs and preferences can differ between patients, needs may vary within patients over time [6]. If we want to better adapt technology features to the needs of patients, we need to account for these changing preferences, needs, and contexts of use. To account for such dynamics, a long-term perspective on eHealth design, as indicated by the CeHRes Roadmap, is preferred and will contribute to the likelihood of sustainable implementation of the health management platforms.

### Goal of This Study

Therefore, the aim of this study was to identify and monitor patients’ needs for support from a web-based health management platform and how these needs change over time. Compliant with the CeHRes Roadmap, we investigated how the Vital10 PHP fits patients’ rehabilitation goals and day-to-day activities. We included user expectations and experiences and actual use data to explain the implementation process of the platform from a user perspective, as the uptake and acceptance of the platform are prerequisites for its implementation. In addition, we considered the domains of the Nonadoption, Abandonment, Scale-up, Spread, Sustainability (NASSS) framework to identify potential factors impeding or facilitating the adoption and continued use of the Vital10 PHP [11]. Although the NASSS framework contains 7 domains in total, only the domains of condition, technology, and adopter were considered relevant to this study. However, in this study, we only included the perspectives of patients (users) as adopters. The findings of our study will provide input for platform redesign and implementation strategies for lifestyle-supporting eHealth tools. In addition, it will show how use patterns will develop over time. We aimed to answer the following research questions: “How do novice and more advanced users experience an online health management platform?*”* “What user expectations support or hinder the adoption of an online health management platform?” and *“*How does actual usage relate to user experiences and adoption?”

## Methods

### Study Design

This mixed methods study combined a 2-round usability study with a log data analysis. The usability study consisted of 2 rounds of web-based usability tests with additional interviews, inspired by the NASSS framework [11]. The usability tests were conducted using a scenario-based think-aloud method [12] and were captured by video and audio recordings. The first round was conducted before the participants used the Vital10 PHP (0-month group), and the second round was conducted after they used the Vital10 PHP for 6 months (6-month group). The first round was conducted between July and October 2020, and the second round was conducted between April and May 2021. Both usability rounds were held on the web via Microsoft Teams (Microsoft Corp) because of COVID-19 restrictions. Conducting 2 different rounds of usability testing in distinct phases of platform use and cardiac rehabilitation enabled us to identify patients’ needs, expectations, and experiences and evaluate whether and how these might change over time. In addition, testing scenarios in 2 different rounds over time enabled us to study the fulfillment of the needs of the patients both prospectively and retrospectively because in the first round, the primary focus was on usability, whereas in the second round, the focus was on engagement and adherence. In addition, in the second round, we included both patients who participated in the first round and new patients. In this manner, the change in participants’ experiences over 6 months (within person) was captured, without the influence of a t0 (study of the 0-month group) assessment. The log data analysis was conducted with a data set from the users of the Vital10 PHP from January 3, 2020, to March 15, 2021 (437 consecutive days). The log data analysis enabled us to study the actual use patterns of the patients over time, which made it possible to compare objective use behavior with the users’ self-reported experiences and intended use as envisioned by the Vital10 PHP developers.

### The Vital10 PHP

**Figure 1:**
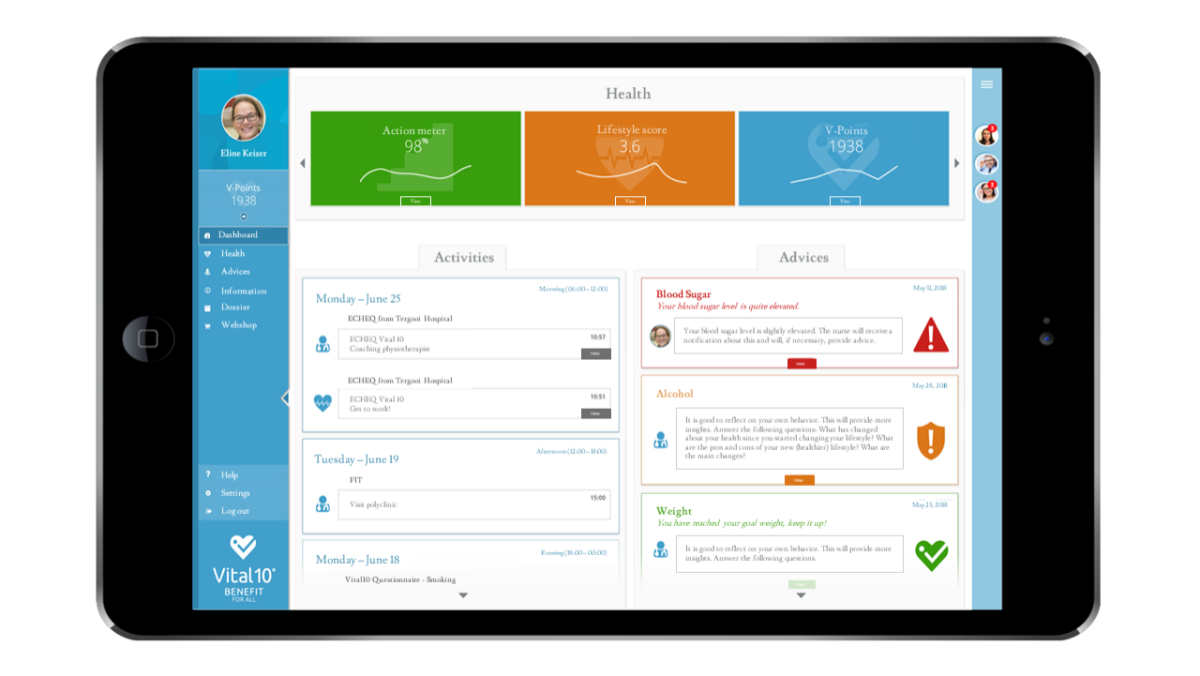
Screenshot of the dashboard of the Vital10 Personal Health Platform.

The usability tests were performed with the Vital10 PHP, a platform developed to support patients with CVD during and after cardiac rehabilitation [10]. The development of this platform was initiated by the BENEFIT consortium, which consists of researchers, cardiologists, general practitioners, eHealth experts, and data scientists. The BENEFIT consortium aims to create a national ecosystem with embedded evidence-based interventions that promote a healthy lifestyle and reward patients for taking actions that contribute to a healthy lifestyle [13]. The intended platform is currently hosted by Vital10, an organization established by a team of multidisciplinary information communication technology and health care professionals who aim to support people with their health [14]. The Vital10 PHP is a dynamic platform in which both patients and health care providers (eg, cardiologist, physiotherapist, dietician, and psychologist), as well as several nonmedical stakeholders such as intervention providers (eg, quit smoking program providers and personal trainers) and loyalty partners (eg, those who provide discount on products), are involved. See Figure 1 for a screenshot of the Vital10 PHP’s dashboard. Additional screenshots of the Vital10 PHP are presented in Multimedia Appendix 1.

The dashboard displays the menu on the left hand (in blue). The green (*Action meter*), orange (*Lifestyle Score*), and blue (*V-points*) blocks are lifestyle modules with the patient’s data. “Activities” shows tasks that the patients have to do on the platform and reminders of visits to health care professionals. “Advices” shows automatically generated advice or personal feedback from a linked professional based on the health- and lifestyle-related data. In the blue border on the right site, chat windows with health care workers (eg, their lifestyle coach) are displayed.

Several persuasive features from the Persuasive Systems Design model [15] were added to the design of the Vital10 PHP (eg, self-monitoring of data, rewards, reminders, and suggestions) to make it more appealing to users and motivate them to improve their lifestyle. In addition, several evidence-based interventions to promote a healthy lifestyle are embedded in the platform. The platform includes different modules, such as weight, blood pressure, alcohol, stress, sleep, and physical activity. The platform is characterized by the provision of rewards to patients for taking actions that contribute to a healthier lifestyle [13]. For example, patients set goals to improve their lifestyle (eg, focusing on increasing physical activity levels or aiming for a healthier diet), and they can log their health data (eg, weight and blood pressure) and behavioral data (eg, step count and daily food intake). Every time patients set goals and log their data, they receive *points*. Saved points can be used in a web shop to *buy* health-stimulating products (eg, diet and lifestyle books and a heart rate monitor) as well as luxury products (eg, hotel trips). In addition, the Vital10 PHP provides features that support patients’ motivation (eg, goal feedback and reminder messages) and provides an overview of medical and lifestyle information. For example, when patients set goals and log data, the Vital10 PHP automatically provides feedback on their progress and offers advice. The automatically generated feedback is based on the cutoff values predetermined by Vital10, based on clinical practice guidelines for cardiac health care. The Vital10 PHP also provides a to-do list with tasks to be performed on the platform and an overview of their historical and future medical appointments. In addition, patients can reach out to a real-life coach to ask questions and for advice via the chat function and video consultation.

### Ethics Approval

The University of Twente’s Ethical Committee from the Faculty of Behavioural, Management and Social Sciences (BCE200180) approved this study.

### Consent to Participate

The participants were informed of the voluntary nature of their participation, and confidentiality was guaranteed. All participants verbally provided consent for participation in a voice recording before the start of the usability session.

### Web-Based Usability Tests With Interviews

#### Participants

Two rounds of usability testing were conducted with patients with CVD. In the first round, patients who had just started or were about to start cardiac rehabilitation and, therefore, had been recently introduced to the Vital10 PHP were included. In the second round, patients with CVD who had started cardiac rehabilitation in the past 6 months and had used the Vital10 PHP during their rehabilitation process were included. For both rounds, participants were recruited via convenience sampling from Vital10 users. Participants in the first round who indicated willingness to participate in future research were approached first. Overall, 40% (4/10) of participants took part in both rounds, whereas most participants (4/12, 33%) were included only in round 1 or 2.

#### Procedure

In the first round, the patients who underwent cardiac rehabilitation at Vital10 were sent digital surveys (“V-cheqs”) within the Vital10 PHP, conforming to the Vital10 procedure. In these surveys, their Vital10 coach added the question of whether the patients were willing to be contacted about participation in a research study. If the patients agreed, their Vital10 coach provided the researchers with the patients’ contact details, and they were contacted by a researcher (BEB) via telephone. In this call, the nature and aims of the study were explained, and if the patients agreed to participate, web-based appointments were scheduled. For the second round, the Vital10 coach added the question of whether the patients were willing to be contacted about participation in this study in the V-cheq that the patients had to fill in at that particular moment of cardiac rehabilitation, conforming to the Vital10 procedure. Establishing contact and scheduling web-based appointments were similar to the first round and were done by another researcher (CS).

Both rounds of usability testing took place on the web via Microsoft Teams because of the COVID-19 restrictions that were in force. For both rounds, the same usability test and interview protocols were used. During the digital meeting, the participants provided verbal informed consent after the recording was initiated by the researcher. After the recording started, the patients were asked in the first part of the meeting to provide information on their demographic characteristics, digital skills, and cardiac health if they were willing to.

In the second part (usability session), several core functions of the Vital10 PHP were evaluated by the participants using scenarios. The scenarios were determined based on the Vital10 PHP Patient Journey (including intended use of the platform), which was developed during brainstorm sessions of the BENEFIT research group and based on adherence and log data literature [16], focusing beyond general use belief of “more is better.” In fact, a more realistic use of the PHP was formulated, considering rehabilitation goals as well as accounting for personal use preferences and personal needs, among other things. For example, the participants were asked to fill in some of the provided V-cheqs, set goals, log (fictive) health data, view their (fictive) progress, and view some of the provided feedback and advice, which were generated automatically by the Vital10 PHP based on the self-monitored data. See Multimedia Appendix 2 for an overview of the scenarios. During the scenarios, the researchers intervened as little as possible because the participants had to show how they would navigate the platform by themselves. They were prompted to think aloud [12]. They could log in via newly made accounts of fictitious users during the test so that they did not have to show their own (medical) data and goals on the Vital10 platform.

In the third part (interview questions), the patients were asked about the uptake and use of the Vital10 PHP in their daily lives. For example, they were asked when the Vital10 PHP should be introduced to inexperienced users, whether informal caregivers should be involved, how they perceive the role of health care professionals within the Vital10 PHP, and what they need to achieve their healthy lifestyle goals in their daily life. After closing the interview, the recording was stopped. Subsequently, the patients were mailed a gift certificate via post to thank them for participating. See Multimedia Appendix 2 for an overview of the interview scheme.

#### Data Analysis

After the usability and interview sessions in round 1, the recordings of the usability sessions were pseudonymized and stored in a secure data server at the University of Twente. These data were accessible only to the researchers involved. The recordings of 3 participants from round 1 were not stored correctly owing to technical errors; therefore, these patients were excluded from the study (round 1 participants 4, 7, and 12). The recordings were transcribed verbatim, and all the transcripts were analyzed by BEB to identify fragments about experiences with the Vital10 PHP and the needs and requirements for the design and implementation of eHealth technology. Relevant fragments were labeled with the main codes “experiences,” “design,” and “implementation” in Atlas.ti (version 9, ATLAS.ti Scientific Software Development GmbH) [17]. The fragments within the main codes were analyzed axially to link fragments to each other and create new subcodes within each main code. The analysis of the first round was performed by BEB before the analysis of the second round. The coding scheme of the first round was revised several times by BEB and JW, and the fragments were reread and recoded if necessary. This coding scheme was also used as a foundation for the extraction of data from the second round. In the second round, data were analyzed by BEB and CS similarly to how data were analyzed in the first round. The coding scheme of the second round was revised several times by BEB, CS, and JW, and the fragments were reread and recoded if necessary.

### Log Data Analysis

#### Data Set

This study used a secondary set of data collected before the beginning of the study (from January 3, 2020, to March 15, 2021; 437 days). All users were invited by their health care professionals to use the Vital10 PHP after they experienced a cardiovascular event. The users had to agree to the terms of use, which included the guarantee that anonymized log data would be used only for research purposes, before they could first access the platform. These log data do not include any demographic or medical data but solely use-related data.

#### Data Analysis

The data set was prepared and analyzed using R (R Foundation for Statistical Computing, version 1.3.1056). The original variables were timestamp, user ID, http method, and apicall. Sessions of use were created when a session lasted for at least 60 seconds. After 30 minutes of inactivity, a new session began. The following new variables were created: session number, total sessions, total time used, days between sessions, lapse, session length, mean total days used, and platform component. See Table 1 for an overview of the variables.

For descriptive analysis, only long-time users were considered. Research shows that many users stop using eHealth within 3 weeks [18]. Any user who used it for >3 weeks was either adherent or nonadherent. To be defined as adherent, we needed a definition for intended use. Members of the BENEFIT consortium envisioned this as follows: *minimum of both once per week log-in and filling in the vitality score*. This was defined by the BENEFIT project team in a brainstorming session, and based on earlier studies in the area of intended use and adherence [16] and the research team’s assessment of the platform and rehabilitation goals. Every user can have some lapse in adherence, as this is common [19]. Therefore, not every user who has 1 lapse is labeled as nonadherent. Users who (1) have too many lapses, relative to their total time using the platform; (2) have 4 weeks of nonuse; or (3) stop using the platform qualify as nonadherent. Consequently, users to whom none of these variables apply are considered adherent. In Table 2, the operationalization of adherence variables is summarized.

**Table 1:** Variables for log data analysis.

Variable	Explanation
Timestamp	Date and time
User ID	Unique user ID
http method	GET^a^ for receiving information from the platform and POST^b^ for posting information on the platform
Apicall	The activity performed
Session number	Count of sessions for a user
Total sessions	Maximum number of sessions a user performed on the platform
Total time used	Total number of days of using the platform
Days between sessions	Number of days between 2 sessions
Lapse	Gap of >7 days between sessions
Session length	The length of a session in minutes
Mean of total days between sessions	The sum of *days between sessions* divided by *session number*, which indicates the average time in days between using the platform
Platform component	Indicates whether a platform component was used; for example, “Advice”=yes explains that the user did use the advice

^a^GET: an action performed by the user on the platform to receive information from the platform.

^b^POST: an action performed by the user on the platform to post or upload information on the platform.

**Table 2:** Operationalization of nonadherence.

Variable	Operationalization
4 weeks of nonuse	Gap of >28 days between sessions
Too many lapses	The total number of lapses is higher than allowed for the “total time used” based on the following formula: “total time used”^a^ 0.034< “lapse”; 0.034 means that one lapse is allowed every 30 days (1 month) Too many lapses = 0.034 total time used<lapse
Stopped using	The last “timestamp” is earlier than February 15, 2021, (4 weeks before the end of the log data) or “total time used”<364 (1 year)

^a^Only one of the variables must be true to be labeled as nonadherent.

## Results

### Usability Tests and Interviews

#### First-Round Sample (0 Months)

##### Participants

In total, 10 participants were included at 0 months. The sample comprised 80% (8/10) of males. The mean age of the patients was 62 (range 48-76) years. The most reported cardiovascular condition was myocardial infarction. The participants reported that they started to improve their lifestyle after the cardiac incident by focusing on maintaining a healthier diet and increasing their physical activity. However, some of them mentioned that they already focused on a healthy lifestyle (mainly diet and physical activity) before the cardiac event. The participants wished to return to the life they had before the event and wanted their anxiety and insecurity about their health condition to be taken away. For example, they indicated a strong feeling of insecurity about their health after they were discharged from the hospital. An overview of the participant characteristics is presented in Table 3.

**Table 3:** Characteristics of the participant sample at 0 months.

Participant number	Sex	Age (years)	Cardiovascular condition
Participant 1	Male	48	Heart surgery
Participant 2	Male	62	Heart surgery
Participant 3	Male	66	Aorta aneurysm
Participant 5	Male	63	Myocardial infarction
Participant 6	Male	70	Arrhythmia
Participant 8	Male	52	Congestive heart failure
Participant 9	Female	76	Myocardial infarction
Participant 10	Male	72	Congenital heart defect
Participant 11	Female	DNS^a^	Myocardial infarction with complications (cardiac arrest or arterial bleeding)
Participant 13	Male	52	Myocardial infarction

^a^DNS: did not state.

##### Expectations and Experiences at 0 Months

The 0-month usability test showed that half of the participants (5/10, 50%) had not started using the platform. They had filled in a few introduction questionnaires, for example, but they did not start exploring the platform by themselves. These participants indicated that they had no need for technological support to monitor their data. The indicated reasons were, for example, that they thought that they already had a healthy lifestyle or that they did not feel comfortable using technology. Others indicated that they were open to being supported by technology. In general, the participants stated that support by a technological platform does not have any obligation and is flexible, which they appreciated because it makes the platform accessible for them:

[Researcher: How desirable is it for you to be supported by a platform instead of a real person?] It is easier. [Researcher: Easier, because?] Because you can use it if you feel like it and want to make time for it. If you are confronted with a real person, then you must make all kinds of agreements, all kinds of obligations, and yes...I hate all kind of obligations. I like that I can use the platform whenever I want.

Participant 6

During the scenarios, the participants noticed that it was interesting to monitor their own health data and view their progress. It was indicated that *watching their progress* would motivate them to adopt or maintain a healthy behavior:

It gives stimulation when you see it descend, the line, which gives extra motivation

Participant 1

In addition, they were positive about the possibility of revising older data and advice, and they appreciated that all their data, appointments, and to-dos were accessible on one page. However, the participants indicated that it is important for the platform to use all health data when providing feedback (eg, historical data). At that moment, they indicated that they only received feedback from the platform on a snapshot of data and that the platform did not consider the progress since the last logged data when providing feedback to the user:

Below you can see all the advice that is currently provided on your input. I have the idea that it is just a snapshot, that advice, and that it does not revise the data history [...] For example, alcohol consumption: every now and then during the week I drink some glasses, but in the weekend, I fill in zero glasses. And then you see ‘congratulations, you’re doing well, you didn’t drink alcohol.’ Then I think, hey, it does not look at the history of the past few days...

Participant 10

There were contradictory attitudes toward the use of reminders. It was mentioned that the frequency of *receiving reminders* was annoying or overwhelming, whereas others indicated that it helped them with remembering what still needed to be done (eg, daily tasks).

##### Needs and Requirements for Support From a Health Management Platform

During the interview sessions, several possibilities of what could be provided on the platform were discussed. For example, the participants wished to *have an incentive (“*a stick behind the door”) to monitor their data, and they appreciated the option to set goals on the platform:

It gives you an overview, but it is also a big incentive to keep measuring your health. Rehabilitation is not just training.

Participant 5

A reward system, such as that provided on the Vital10 PHP, was generally considered undesirable or unnecessary by the participants. They stated that gaining or regaining their health was their aim, not receiving rewards. One of the participants also indicated that the platform should provide fitness exercises or other tips for physical activity for this specific patient group. In addition, the participants preferred *personalized advice.* For example, although a participant had already lost considerable weight, he or she was still too heavy. Therefore, the platform provided the feedback that this person should lose weight. This was very demotivating for the participant because he or she was already trying hard, and it would have been appreciated if the progress he or she made was also reviewed and mentioned.

Well, you know...at some point you had a heart attack and then you are sent home. And then you receive rehabilitation guidance, because you must start exercising again and regain confidence. So, then I do not pay attention to getting gifts. I think those goals, my health goals are sufficient for me to be motivated to start, do you understand?

Participant 5

The participants indicated their need for visualization of their data. Insight into their own data and progress was experienced as an added value. For example, the use of colors or graphs makes it immediately clear what the current health status is. However, it was also noted that this could be confrontational. The advice the platform provides based on health data was interesting for some participants, whereas others mentioned that it was patronizing and that they did not need advice from a platform or person other than their health care professional:

I clicked once on it [Provided advice] and it was a bit patronizing. And very brief, considerably basic information.

Participant 3

The participants appreciated that the platform provided additional information about their disease and how to change their lifestyle. They indicated that sometimes, there was no time to discuss this with their health care professionals during appointments or that they were afraid to ask questions:

[Researcher: What do you think about this kind of information being here?] It is incredibly wise, because people see upper and lower blood pressure...usually, you have to be quiet during the doctor’s examination and then you do not ask what those different things are. So, I like that it is explained in here

Participant 10

The participants indicated needing *reliable information* on the platform or a reference to another reliable source. It was mentioned that the information provided should be concise because an overload of information can be overwhelming. In addition to the information in the text, they also wished to have direct and personal contact, for example, for asking questions without urgency.

The participants mentioned *that it was not clear what role health care professionals* play on the platform. They indicated that they wanted their health data on the platform to be visible to health care professionals. They thought that it would be useful if they can show their logged data to, for example, their cardiologist during appointments. However, it was also mentioned that the participants did not feel comfortable with *sharing health data via technology,* but they suggested that they could show their data easily to their health care professional if they could print an overview of their progress. These participants appreciated logging data such as body weight and blood pressure on the platform but did not want all their medical history to be shown (such as prescribed medication, medical incidents, and previous appointments). In addition, they expected health care professionals to *contact them after their logged data becomes* “red” (eg, if high blood pressure is too high, the module will be colored red) or for the platform to automatically alert the health care professional in such a case:

There should be a notification system that if, for example, your blood pressure is an increasing pattern, then the cardiologist receives automatically a notification with “that patient is not doing so well.”

Participant 8

##### Needs and Requirements for the Adoption of a Health Management Platform

Several other needs and requirements were indicated to help patients adopt the platform in their daily lives. The participants mentioned that they need the *platform to be introduced* and explained to inexperienced users while they are still in the hospital (before discharge). It should then be clarified *for how long the use of the platform* is expected and what the intended use is. They indicated that the strategy of implementation should be tailored to different target groups because different patients *might have different digital skills*. For example, the participants indicated that they feel uncomfortable with only digital support and preferred a combination of digital support and real-life support:

There is a distinction between the younger, middle-aged, and older patients. I can imagine that there are some older people who have a little more trouble using a platform, and there will be, for example, a little more guidance desired.

Participant 1

A *combination of support by health care professionals and a technological platform* is appreciated because this is perceived as a more personal approach. In addition, the participants thought that *an app would fit better in their daily life* than a web page. They also preferred to *connect measuring equipment* (eg, Fitbit [Fitbit Inc] and iWatch [Apple Inc]) to the platform to automatically monitor data. The participants indicated that they did not need their family or friends to be involved on the platform.

#### Second-Round Sample (6 Months)

##### Participants

In total, 12 participants were included at 6 months. The study sample comprised 92% (11/12) of males. The mean age of the patients was 59 (range 48-74) years. The most reported cardiovascular conditions were myocardial infarction and cardiomyopathy. All the participants reported that they started improving their lifestyle after the cardiac incident by increasing their physical activity and mainly by focusing on a healthier diet, for example, eating less salt, unhealthy fats, and red meat and consuming more fruit and vegetables. In addition, the participants mentioned that they also focused on reducing stress (factors), increasing their quality of sleep, lowering their alcohol consumption, and maintaining a healthy weight. An overview of the participant characteristics is presented in Table 4.

**Table 4:** Characteristics of the participant sample at 6 months.

Participant number	Sex	Age	Cardiovascular condition
Participant 1	Female	DNS^a^	Congenital heart defect
Participant 2^b^	Male	63	Heart surgery
Participant 3^b^	Male	64	Myocardial infarction (2 times)
Participant 4	Male	66	Myocardial infarction (3 times)
Participant 5	Male	63	Heart surgery
Participant 6^b^	Male	49	Heart surgery
Participant 7^b^	Male	58	Myocardial infarction
Participant 8	Male	74	Preventive vascular surgery + stent
Participant 9	Male	48	Myocardial infarction with complication (cardiac arrest)
Participant 10	Male	48	Cardiomyopathy
Participant 11	Male	64	Cardiomyopathy and multiple myocardial infarctions
Participant 12	Male	52	Inherited cardiac conditions and angina pectoris

^a^DNS: did not state.

^b^These participants were also included in the round 1 sample (0 months).

##### Expectations and Experiences at 6 Months

After using the platform for multiple months, the participants stated that it was motivating and easy to log data. The colors and signs provided *a direct and clear overview of data* (Figure 1). Tracking data and progress stimulated and motivated the participants, and *setting goals made them more aware of the focus on their lifestyle*. However, the participants mentioned that it was a challenge to get all the lifestyle values on the dashboard to turn “green” (eg, in case of a healthy weight or blood pressure, the module will become green). The chat is *easy and accessible for quick contact* with a health care professional, although some participants preferred personal contact. However, the reward system was not valuable to most participants. They indicated that rewards motivated them to log data, but most participants mentioned that they must *be intrinsically motivated to improve their health, not to gain rewards*. In addition, some advice and information on the platform were impersonal or basic, which gave the participants a bad feeling. It was also noted that there was a lack of support at the beginning of their use of the system, which was demotivating for them:

Then I get the feeling that it is a general story and not specifically intended for you.

Participant 6

##### Needs and Requirements for Support From a Health Management Platform

A platform should have a *calm and consistent appearance*, and features should be prominently placed and easy to find. In addition, the participants wanted the option to *save interim data while logging, performing tasks, or filling in questionnaires*. They also preferred to review and adjust their data after saving it. They indicated that setting goals helps them improve their lifestyle step by step. It was mentioned that they would prefer it if they could *set several goals at the same moment*, for example, not only diet-related goals but also exercise and quit smoking goals. They wanted the platform to *provide information about a healthy lifestyle*. They needed an incentive (“a stick behind the door”) to adopt and maintain new, healthy behaviors. In addition, they mentioned that they wished to have *peer-contact* on the platform:

I would not be into having contact with other heart patients because treatment and recovery greatly differ between conditions. It would be interesting though to read about how other patients deal with experiences of limitations or changes in lifestyle.

Participant 5

The provided information should be complete, correct, and personalized to the user’s needs and interests. The participants noticed that the current information and advice were not applicable to them. They indicated that they will be more motivated to use the platform if the *information and advice were applicable and reliable*. In addition, they indicated that the information was not inspiring:

I also kept track of my data for a while, but that slowly stopped because I was not necessarily happy with all the advice I received, or just the advices I did not receive, so that’s why.

Participant 9

The participants indicated their wish for *feedback not only on self-monitored data* but also on their activities. This was especially true during the COVID-19 pandemic (because of the lockdown, there was no real-life physical activity rehabilitation) when the participants *missed receiving feedback* on their physical performance, and they needed some reassurance about what their body was capable of doing.

The participants needed an *overview of all their medical health data*. Although it was mentioned that a commercial platform is not the right place for saving medical data, most participants indicated that they wanted to exchange data with health care professionals. Some want to make their data *accessible for the health care professional* on the platform, whereas others wished to show their logged data on the platform by themselves during appointments:

Well, it is useful if all medical data is put together, but I do not know If this platform is the right place. I do not know whether a commercial organization such as I see this [Vital10 PHP]...whether I would consider that as the right place. I would prefer to have that at for example my general practitioner or the hospital.

Participant 9

##### Needs and Requirements for the Adoption of a Health Management Platform

The Vital10 PHP participants indicated that one of the requirements for the adoption of the platform is that it should be *introduced and explained before their discharge from the hospital* or at the start of the (live) cardiac rehabilitation:

Nowadays you are no longer in the hospital for ten days, before you know it you are home again. And then? Then it is especially useful if you get it from the hospital, go look for it, you are working on it, you can give it a place, you can describe it in your goal, so you can look back on what did I do wrong. Of course, it does not have to, it can also just be a physical thing. You will be helped a bit with that and triggered to think about it, but you can also do something with it. Otherwise, I will come home and then there will be nothing, yes, continue to live happily, but at least that is what I experienced from cheerfully to live on, there are still some steps needed.

Participant 3

They suggested this timing of introduction because they mentioned that it was important that patients or users be informed in person about the platform and *have the possibility of asking questions or receiving help while using it*. For less digitally skilled participants, it was difficult to understand how to use the platform or what was expected from them. Moreover, the participants indicated that the platform should be provided in addition to usual cardiac care. They mentioned that the platform supports them, but they wished that it be implemented not only as an addition to cardiac rehabilitation but also as an addition to regular (cardiac) care. It should support them but should not replace usual care or personal contact with health care professionals.

The participants indicated that it was irritating that it was not clear whether the platform could interoperate with other measurement equipment, such as Fitbit or iWatch. They were bothered by the fact that they had to log the data manually on the platform and preferred an automatic synchronization of these data. In addition, the participants indicated that they need to receive triggers for using the platform. The current reminders sent by the Vital10 PHP were helpful for some of the participants, although others thought that these were irritating and would like the possibility of changing the settings related to the frequency of receiving reminders:

I think it [Vital10 PHP] should trigger usage...The rehabilitation trajectory is 6 weeks, and the platform could for example after 10 or 12 weeks send you a notification asking: “How are you now?” Or not even a question, but just a notification which triggers you to look at the platform and fill in some data. The trajectory stops after 6 weeks, period. Then you must do it by yourself. That is true, but I still have questions after 10 weeks...

Participant 3

### Log Data Analysis

#### Use Statistics

A total of 762 users were invited to use the platform. Of these, 69.6% (506/762) of users were long-term users (>3 weeks of use) and were thus included in the analysis. In total, 10,285 sessions were performed, of which 9606 (93.4%) sessions were performed by nonadherent users. On average, it took the users 14 minutes per session. This average session length decreased from 37.6 minutes for the first session to 11.5 minutes for the eighth session and beyond. Half of the users (300/506, 59.2%) quit using the platform during the first 11 sessions. Over 49% of the users had a total number of sessions that represent at least 1 session per week. The platform was most used between 9 AM to 12 PM, with an average gap of 6.5 days between the sessions. Table 5 provides an overview of the general use statistics.

**Table 5:** General use statistics by long-term users (n=506).

	Average	Range
Number of sessions	19^a^	2-283
Session length (minutes)	14^a^	1-241
First session length (minutes)	37.6^a^	1-241
Length of the eighth session and beyond (minutes)	11.5^a^	1-113
Total time used (days)	100^a^	21-381
Lapses	2.7	0-15
Mean total days used	110	21-392

^a^Variables are averages.

#### Long-term Use Pattern of the Vital10 PHP

**Figure 2:**
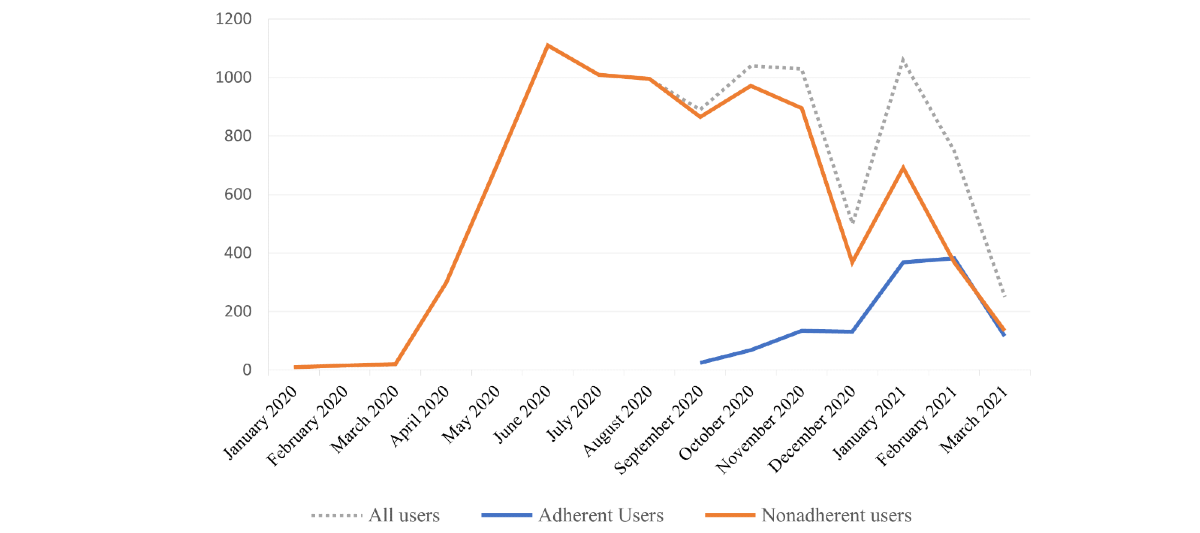
Long-term use of the Vital10 Personal Health Platform by all the included users.

Over the entire study period (437 consecutive days from January 3, 2020, to March 15, 2021), each patient visited the Vital10 PHP an average of 19 times, for approximately 110 days (range 21-381 days). An overall decline in use was observed over time (Figure 2). Most sessions were performed by nonadherent users, who make up 93.4% (n=712) of all users.

#### Long-term Use Pattern of the Components of the Vital10 PHP

The platform consists of different components. Table 6 shows the different components and reports the number of users who used these components at least once.

**Figure 3:**
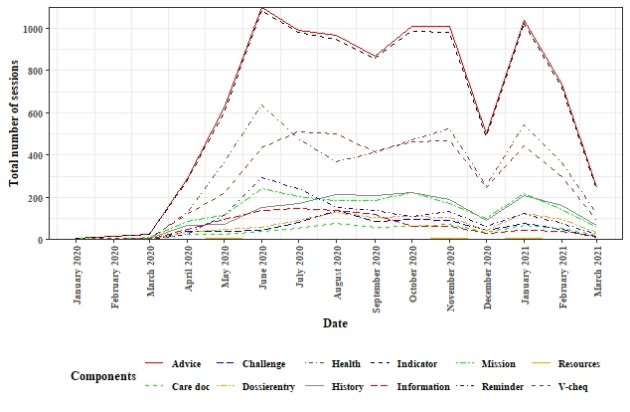
Long-term use of the different components of the Vital10 Personal Health Platform by all the included users.

Figure 3 displays the long-term use patterns of the different Vital10 PHP components for all the included users. The components “Advice” and “Indicator” were used the most by both adherent and nonadherent users, followed by “V-cheq” and “Health.” “Care doc” and “resources” were used rarely or not at all used by both groups.

**Table 6:** Platform component use.

	Total number of users who visited the component at least once, n (%)		If yes, the percentage of sessions the component was used in
	Yes	No	
Advice	506 (100)	0 (0)	98.2
Challenge^a^	452 (89.3)	54 (10.7)	9.2
Mission^a^	408 (80.6)	98 (19.4)	22.9
Health^b^	331 (65.4)	175 (34.6)	56.4
Record^c^	322 (63.6)	184 (36.4)	11.5
History^c^	432 (85.4)	74 (14.6)	21.5
Care doc^c^	233 (46)	273 (54)	6.8
Indicator^d^	506 (100)	0 (0)	96.4
Information^d^	267 (52.8)	239 (47.2)	10.8
Resources	13 (2.6)	493 (97.4)	0.3
Reminders	200 (39.5)	306 (60.5)	18.3
V-cheq	499 (98.6)	7 (1.4)	47.6

^a^Challenge and mission refer to goal setting.

^b^Health refers to self-monitoring.

^c^Record, history, and care doc refer to medical records.

^d^Indicator and information refer to information.

## Discussion

### Principal Findings

The aim of this study was to identify and monitor patients’ needs for support from a web-based health management platform and how these needs change over time. On the basis of these findings, we can conclude that after the start of cardiac rehabilitation, a health management platform can support patients with CVD in adopting and maintaining a healthy lifestyle by helping them self-monitor data, watching their progress, and be (kept) motivated and reminding of their personal goals. The lack of continued tailored or personalized advice 6 months after cardiac rehabilitation made the platform appear less useful to patients. We noted that most patients easily learned how to use the platform and were interested and motivated by logging their data and viewing their progress. After months of using the platform, patients learned and understood how to use the functions on the platform; None of the usability issues hindered them gravely, nor were they a reason to quit using the system. However, soon after the cardiac rehabilitation program stopped (after approximately 3 months), the use of the platform declined, or patients even quit. Earlier studies showed a similar decline in adherence to supporting technologies in cardiac rehabilitation during the first 3 to 6 months [20,21], and approximately 90% of the patients quit using the technology within a year [22]. However, other studies that used eHealth as follow-up after regular cardiac rehabilitation (eg, with features such as reminders and a personal approach) showed positive effects on adherence to the technology [23-25].

An interesting finding of this study pertains to the incentive system included in the Vital10 PHP, which aims to motivate patients to achieve their goals. The need for “having a stick behind the door,” an overview of personal health data, and receiving personalized care were earlier identified as values for patients with CVD [6]. Moreover, in earlier research within the BENEFIT consortium [26], patients indicated that they want to be extrinsically motivated (eg, with rewards) to accomplish goals. Providing feedback and a personalized approach seem to have a positive effect on adherence [27-29], and our study confirms that a lack of personalization discourages the use of the platform. However, in contrast to earlier findings, our study showed that a reward system is generally not regarded as valuable by patients. In the current version of the Vital10 PHP, most of the implemented rewards were luxury items (eg, handbags and discounts for holiday trips). However, these incentives seemed inappropriate to the patients. These incentives were insufficiently linked to the core value of regaining health. However, we can confirm that patients need a positive trigger or reward because they claimed that their reward was becoming healthy again. In this sense, the regained health that they experience is the positive trigger or reward. This finding provides the insight that patients may initially have a need for extrinsic motivation in the short term; however, in the long term, a shift to focusing on intrinsic motivation is needed to support them. Nevertheless, becoming healthy again is a long-term effect, and a lack of short-term effects can demotivate patients [30-32]. Therefore, it is recommendable to focus on health-related rewards (eg, products in the web shop) to contribute to positive health-related changes (promote a healthy lifestyle). Earlier research showed that using eHealth can improve levels of confidence and self-efficacy in a brief time track in comparison with usual rehabilitation programs [23,33].

Most Vital10 PHP users did not use the platform as intended by the BENEFIT research group. However, this does not necessarily mean that the users did not benefit from the platform. The study showed that patients visit the platform and perform the tasks that are assigned to them. However, as soon as the coaching support stops and the patient has to continue independently (eg, use the functions by themselves and seek support by themselves), the platform use declines. We question whether it is a negative outcome that patients quit using the platform at a certain moment. Quitting the platform does not directly mean that the platform is not functioning well or that patients no longer focus on lifestyle improvement. However, it provides insight into the extent to which patients need support at this stage of improving and maintaining their lifestyle. For example, if patients quit using the platform, it could be possible that the platform was successful in providing the support the patients needed to acquire the skills necessary to improve and maintain a healthy lifestyle and is, therefore, no longer needed. By contrast, although patients might think that they no longer need technological support, previous research shows that a sustainable change in (health) behavior after implementing interventions is limited [34,35]. In the latter case, quitting the platform would be a negative result because it would mean that the platform was not successful (yet) in teaching the patient how to maintain their changed behavior. Therefore, more extensive research is needed into how health and health behaviors change after cardiac rehabilitation with the help of the platform. Future research should identify whether patients who discontinued using the platform were, indeed, capable of maintaining a healthy behavior themselves or whether they missed a personal component or supporting guidance in the longer term.

### Recommendations

Currently, we see that the focus of researchers, developers, and health care institutions is on building a guiding and supportive relationship with patients and on encouraging patients to sustain the use eHealth technologies to improve their health. However, it should be kept in mind that the key to success should not be user adherence to an eHealth technology but adherence to healthy lifestyle habits. Therefore, we recommend not focusing on improving adherence to eHealth as a goal in itself but rather focusing on fulfilling the patients’ values: achieving a healthier lifestyle in real life. eHealth is not a stand-alone support but should be integrated into daily life and treatment processes. Therefore, “off-boarding” from a platform should be encouraged if it helps patients independently and sustainably adhere to their healthy lifestyle habits. In this regard, eHealth can still play a role in the patients’ lives as (back-up) support, especially in cases of relapse or health-related questions, with a focus on helping the participants live healthier lives and making them aware of the products and services in the neighborhood that can support them.

Consequently, if we assume that this “off-boarding” is a transition from needing support from an eHealth technology to using the technology as a back-up, how will we be able to identify or monitor this transition? What will happen to the patients after this transition? Will they no longer identify themselves as patients? How and to what extent can or should we (still) support them? In particular, less is known about how technology can support patients in this transition from short-term lifestyle changes to long-term maintenance [3]. We suggest that future research focus on this “off-boarding” transition and how the insights derived in this regard can be taken into account in the design and implementation of an eHealth technology. In addition, use patterns provide insight into which content or functionalities of an eHealth technology are used and could, therefore, identify when, and to what extent, patients’ needs are fulfilled. From a methodological perspective, it is interesting to note that this study included a mix of participants who took part in only the 0-month or the 6-month iteration as well as participants who took part in both iterations. It remains to be tested in future research what sampling approach best suits a multi-iterative user evaluation.

We want to emphasize the importance of conducting multi-iterative user evaluations that conform to the CeHRes Roadmap. It enabled us to identify changes in the users’ needs and contexts of use, and continuously evaluating the eHealth technology enabled us to respond to these changes in the design or redesign and implementation of the technology. The NASSS framework is complementary to this because it provides guidance on what aspects need to be considered for successful implementation. Thus, the NASSS framework helps focus on what should be done, whereas the CeHRes Roadmap defines how, when, and where. However, in this study, we focused on development and implementation from an end-user perspective. eHealth is not a stand-alone tool, and its integration within daily life and health care will involve multiple stakeholders other than patients as well as a business plan [6]. Therefore, future research should also focus on a more ecological implementation of eHealth by considering the other domains of the NASSS framework, such as additional adopters (eg, other stakeholders such as health care professionals), organizational factors (eg, working routines and capacity to innovate), and the wider system (eg, political, regulatory, or legal processes).

### Strengths and Limitations

A strength of this mixed methods approach is that by performing both a log data analysis and usability tests with interviews, we were able to collect details on the users’ (patients’) experiences with a web-based health management platform and their perspectives on the needs and requirements for the platform and its implementation. We were able to complement this with the data on the actual use of the platform. This allowed us to identify the use patterns of adherent and nonadherent users; furthermore, it could help explain why users quit at certain moments of time or why they do not use certain features (anymore). In addition, conducting 2 rounds of usability testing with interviews enabled us to see differences in the needs and requirements for receiving support from a web-based platform over time. During the first round, the cardiac event had just occurred, and participants may have indicated short-term needs based on their anxiety or uncertainty. During the second round, they had probably processed the cardiac event and already adjusted their lifestyle, which could have given them the opportunity to focus on the long-term needs.

This study also has some limitations. Owing to the COVID-19 pandemic, multiple restrictions were implemented in the Netherlands. Especially during the 0-month iteration, the first month after the COVID-19 outbreak, the cardiac rehabilitation centers of Vital10 were shut down and restricted to web-based care only. The participants were offered different rehabilitation programs (with fewer or even without physical appointments), which might have affected their motivation or willingness to use the platform. Owing to the COVID-19 restrictions, we also had to adjust our recruiting and study procedure to conduct them on the web instead of offline. Considering that our target group just had a life-changing cardiac event during a pandemic, it was quite difficult to include patients during this part of our study. In this qualitative study, we focused on the how and why of platform use and not on quantitative results. However, although we included 10 patients, instead of 12, in the 0-month iteration, we observed data saturation in the interviews. In addition, selection bias may have occurred and caused a more homogenous participant group. Most participants were relatively young compared with the general CVD population. However, by including relatively young patients as well as older patients with CVD, we were able to include both more and less digitally skilled participants. In addition, considering privacy protection, during the recruitment, we as researchers only received the contact details of patients who indicated willingness to be contacted by us. Therefore, the response rate of our recruited sample was unknown.

### Conclusions

We can conclude that the support approach of health management platforms should be personalized: there is no one-way solution, and eHealth is not a stand-alone tool. In the short term, it is important to provide supporting tools to patients because they need to learn how to improve their lifestyle and to feel safe and secure about their health again. However, in the long term, the focus should not be on user adherence to the eHealth technology but on adherence to the values of patients for which the eHealth technology was initially developed, such as having healthy lifestyle habits. Although the underlying core value of platform use is becoming healthy, the more practical needs for which patients (or their context of use) require support from the technology may change over time. Hence, quitting the use of eHealth does not directly mean that the technology is not functioning well or that the patients no longer focus on achieving their value. It could mean that their value is fulfilled or that current content or features of the technology do not contribute anymore to supporting them in achieving a healthy lifestyle. This emphasizes the importance of conducting multi-iterative evaluations to continuously examine whether the technology still meets patients’ need for support to achieve their value. These evaluations enable developers to respond to the changing needs in the design or redesign and implementation of eHealth. Therefore, the implementation of eHealth should also include the transition to a stage where patients might no longer need support from the eHealth technology to achieve and maintain a healthy lifestyle and might be independently and sustainably adherent to their healthy lifestyle habits. Future research should focus on how this transition can be identified and monitored and how these insights can be considered in the design and implementation of the technology. However, in this study, we focused on development and implementation from a user perspective. eHealth is not a stand-alone tool, and its integration within daily life and health care will involve multiple stakeholders other than patients as well as a business plan. The NASSS framework also aids in determining which other stakeholder perspectives as well as organizational or legal aspects of implementation need to be considered for the successful implementation of eHealth technology.

## Appendix

Multimedia Appendix 1 Screenshots and core attributes of the Vital10 Personal Health Platform.

Multimedia Appendix 2 Usability test and interview protocols with scenarios.

## Abbreviations

CVD: cardiovascular disease
NASSS: Nonadoption, Abandonment, Scale-up, Spread, Sustainability
Vital10 PHP: Vital10 Personal Health Platform

## Data Availability

The transcribed data are not publicly available because of privacy restrictions but are available from the corresponding author upon reasonable request.
