# Supplementary figures and images for: Implementation and User Evaluation of an eHealth Technology Platform Supporting Patients With Cardiovascular Disease in Managing Their Health After a Cardiac Event: Mixed Methods Study
Source: JMIR Cardio. 2023 Mar 24;7:e43781. doi: 10.2196/43781 (PMC10131764; doi:10.2196/43781)

## **1. Screenshots and core attributes of the Vital10 PHP**


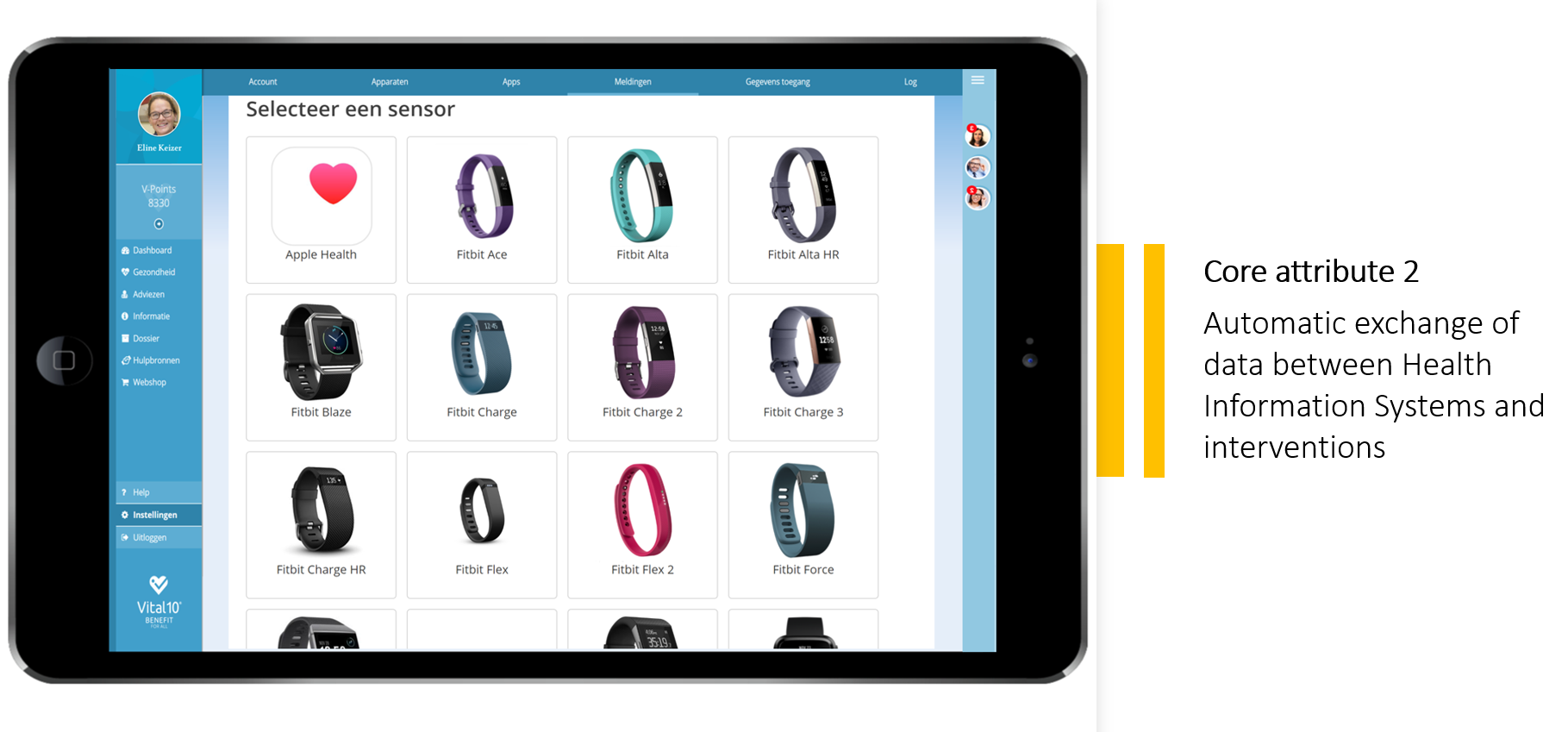

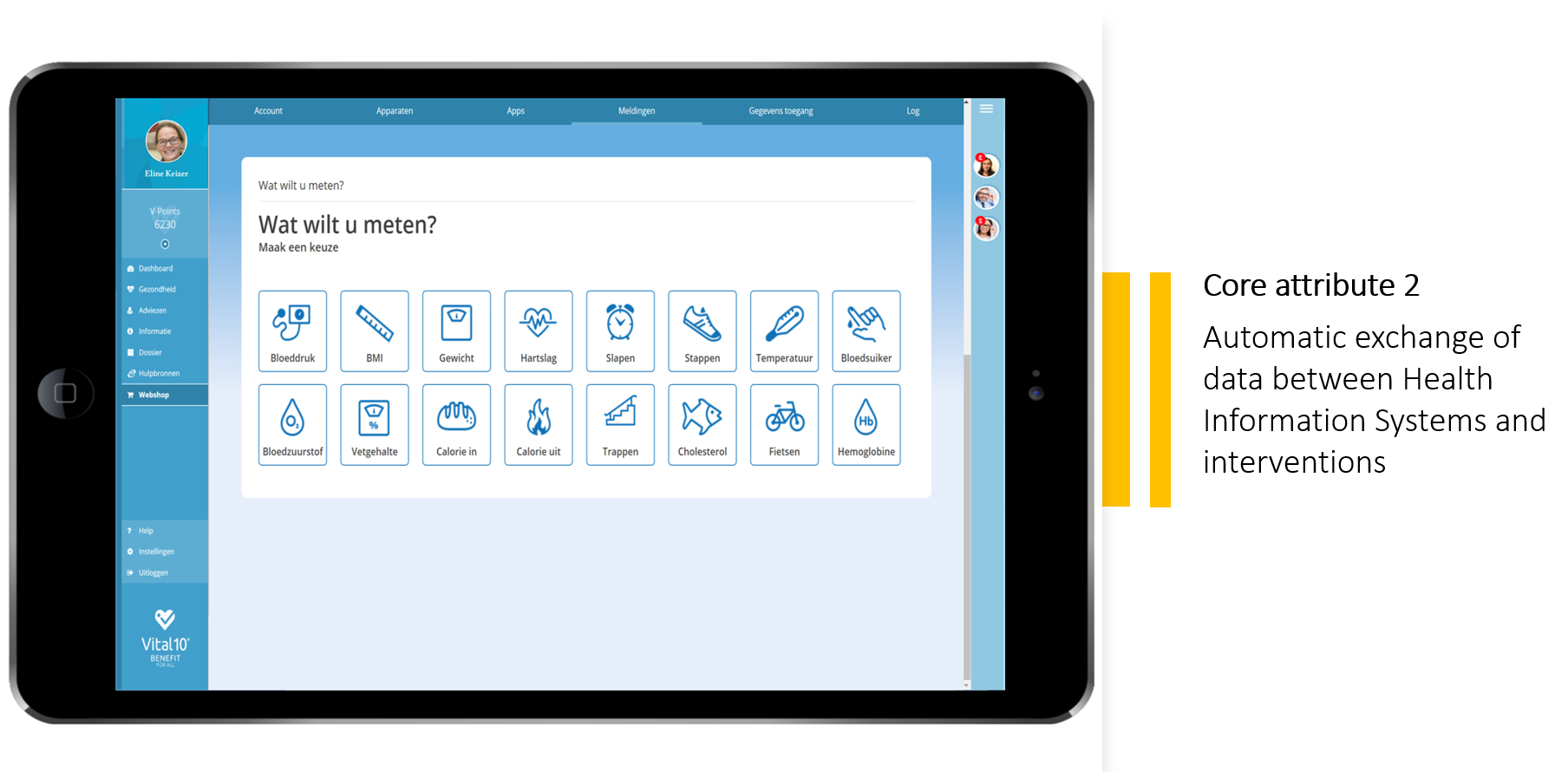

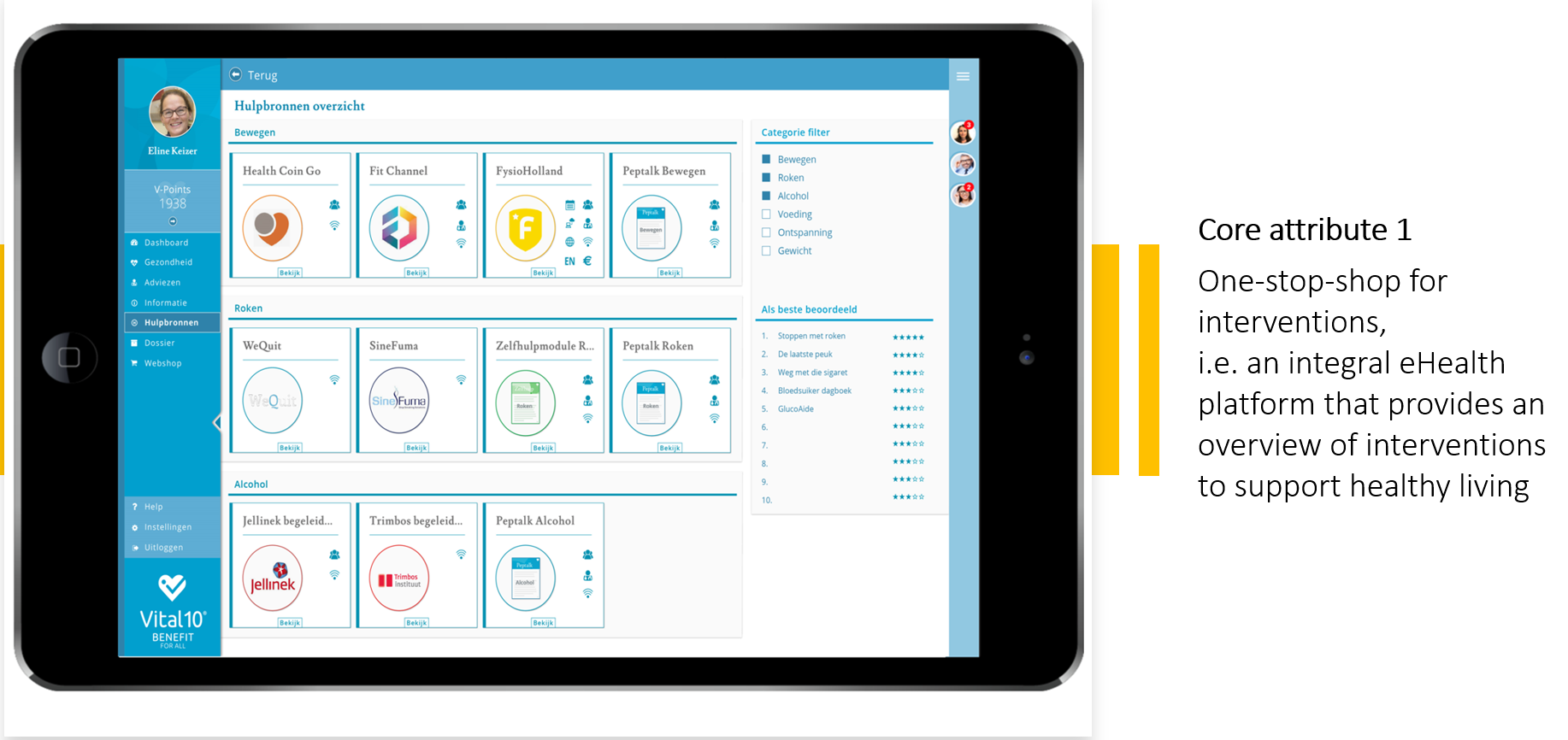


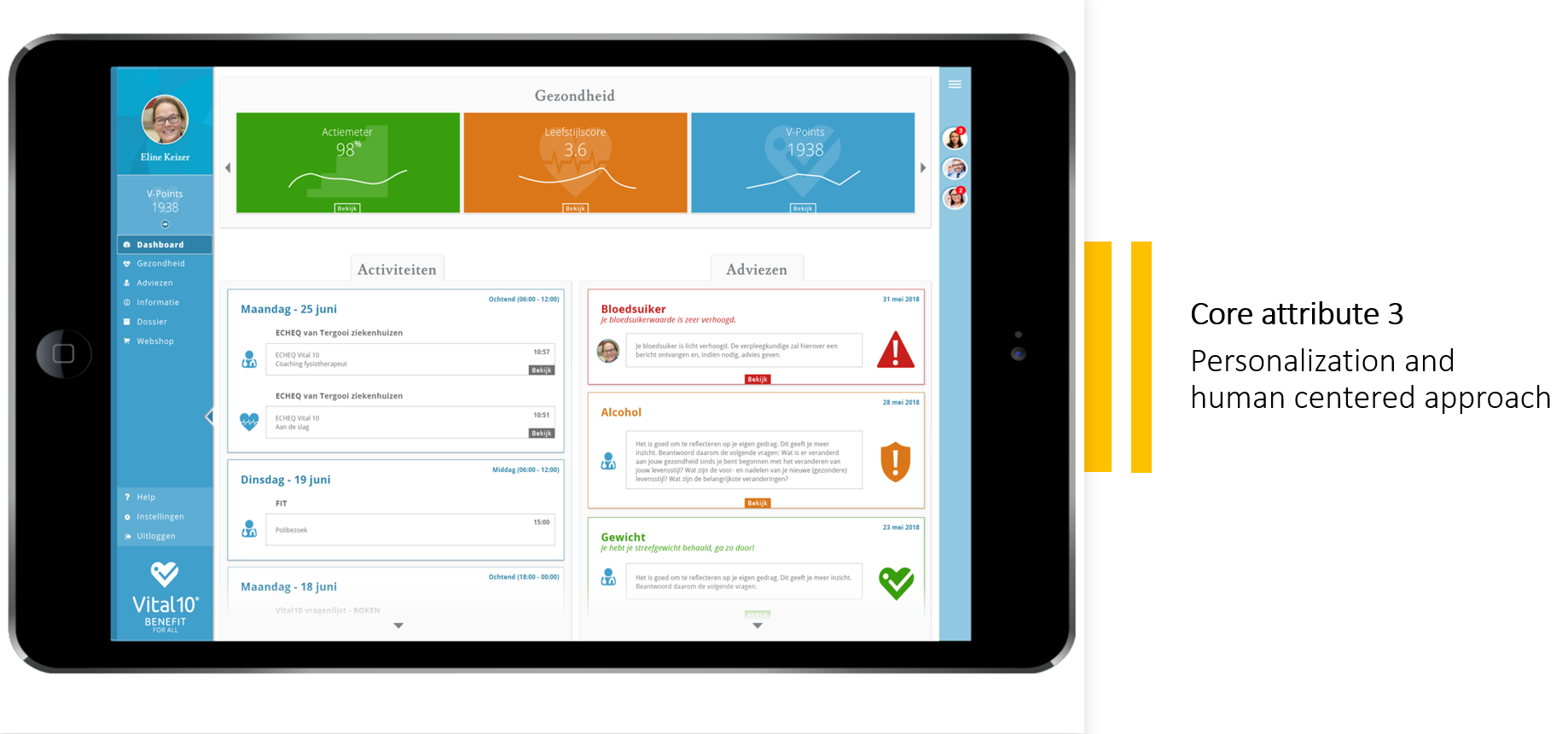


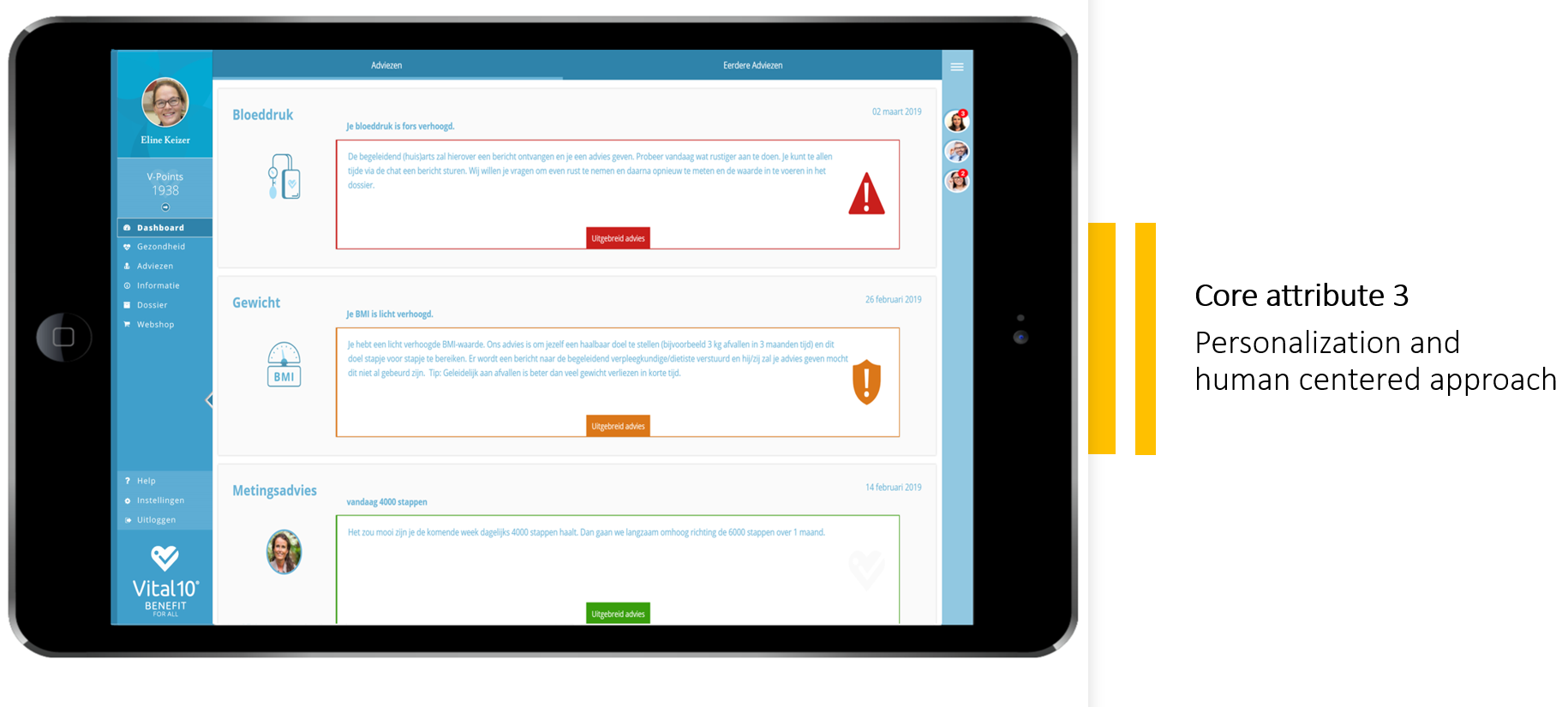


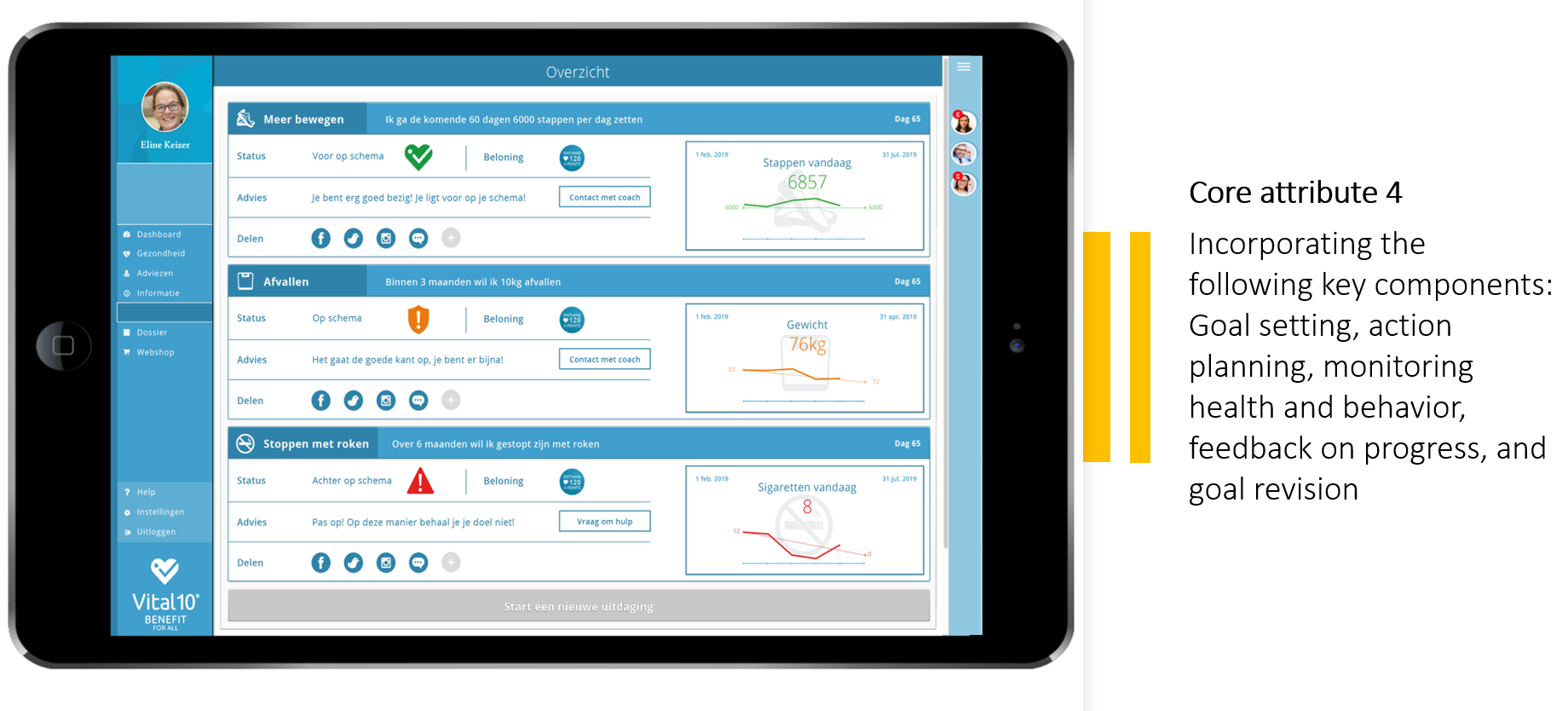


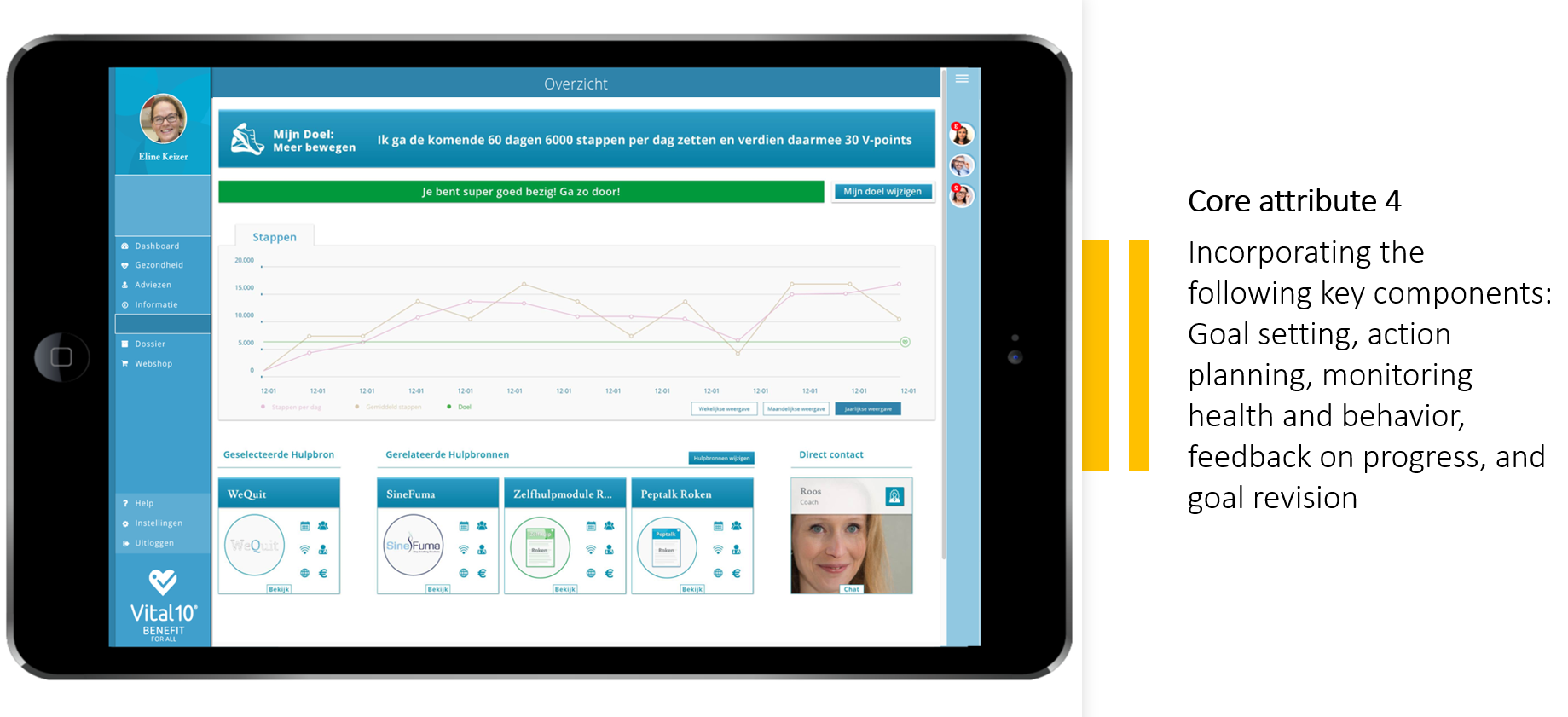


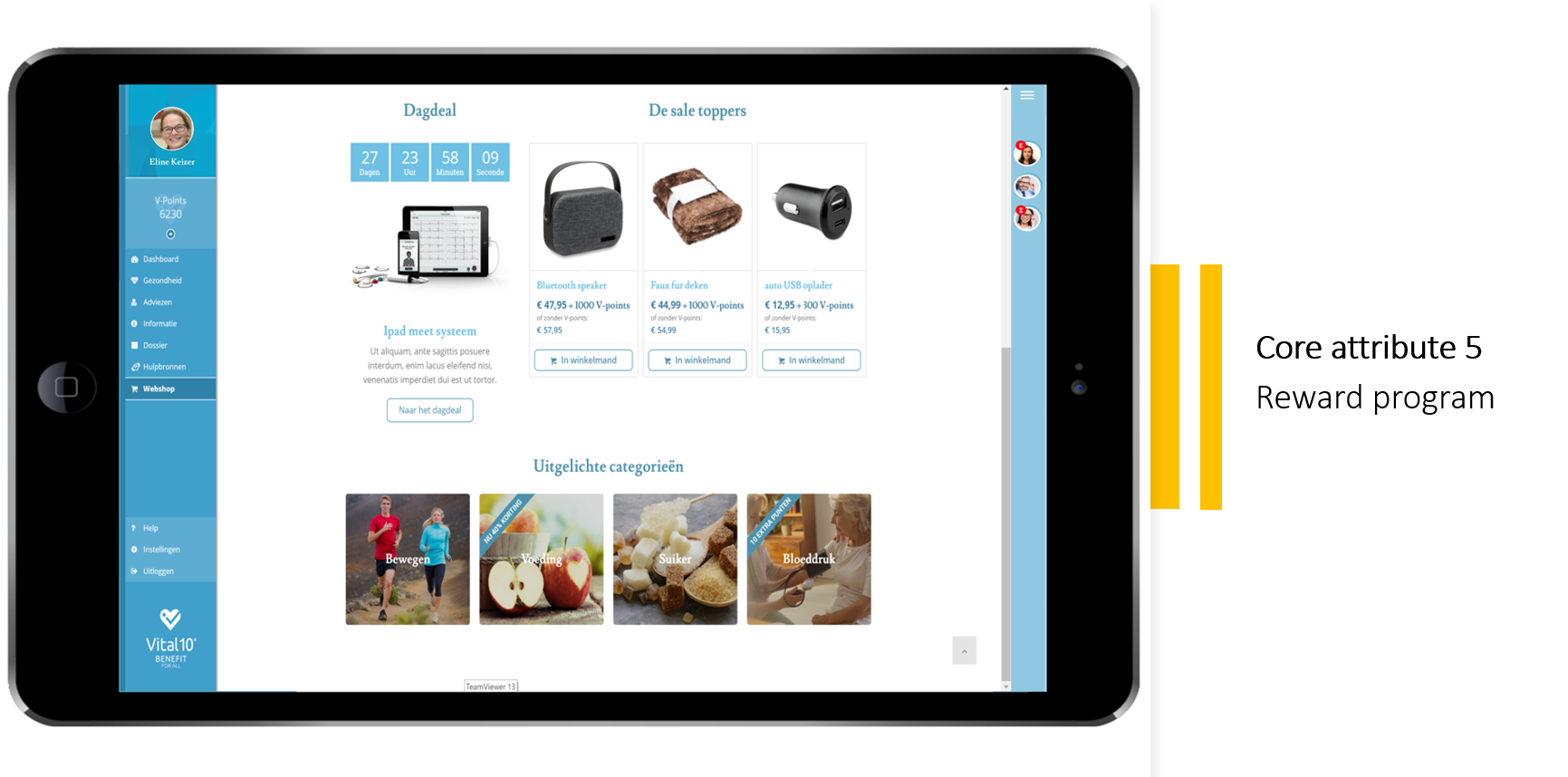


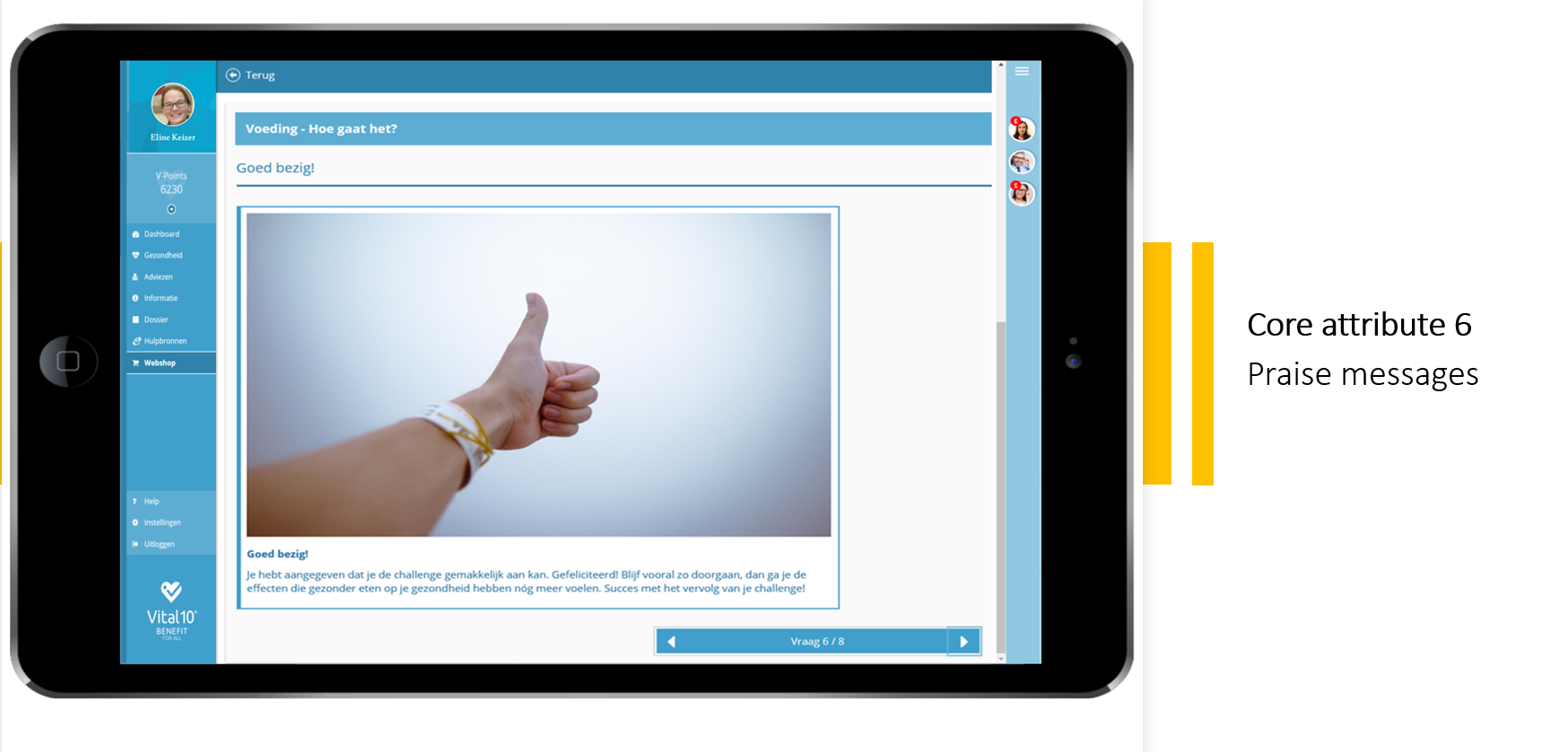


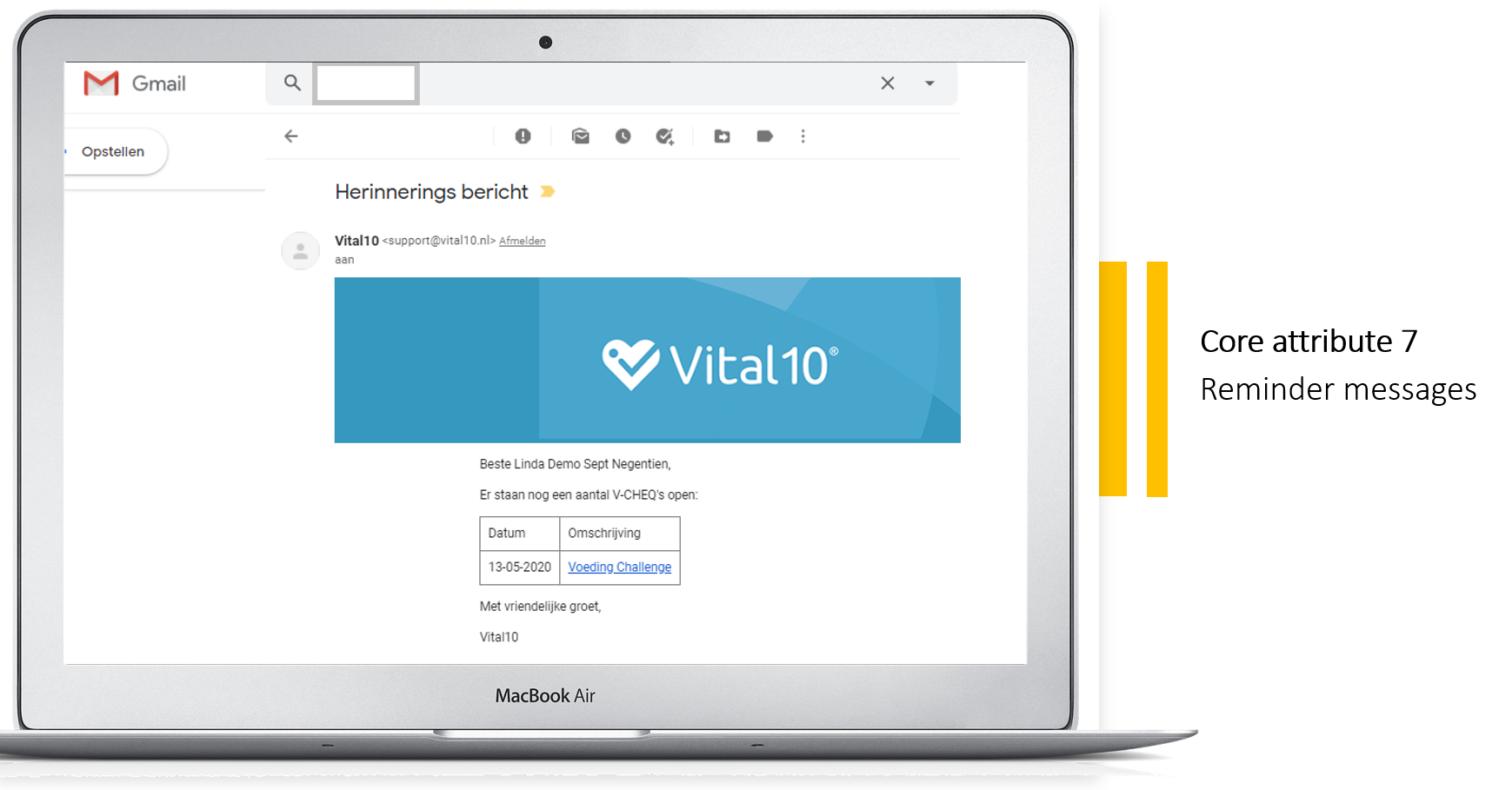


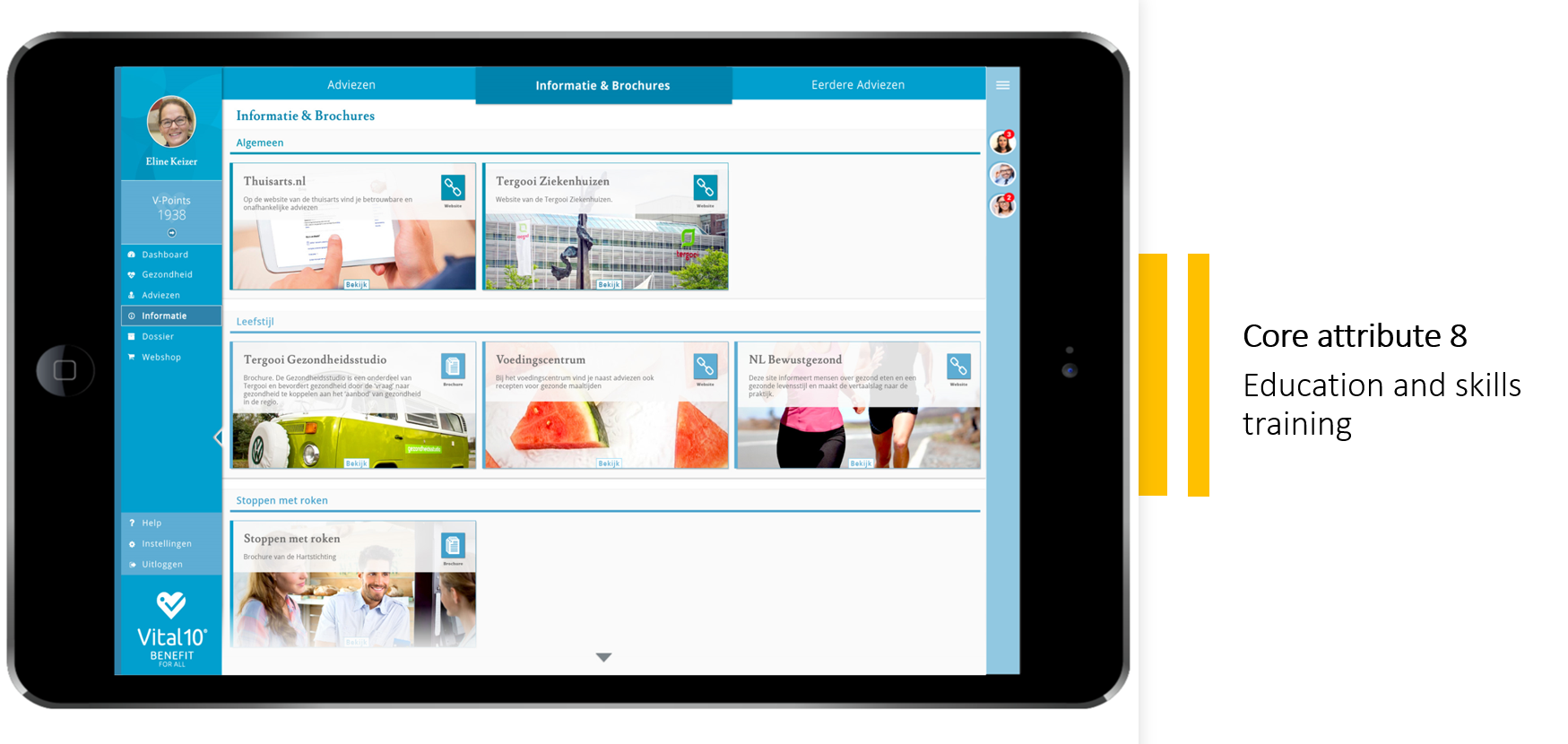


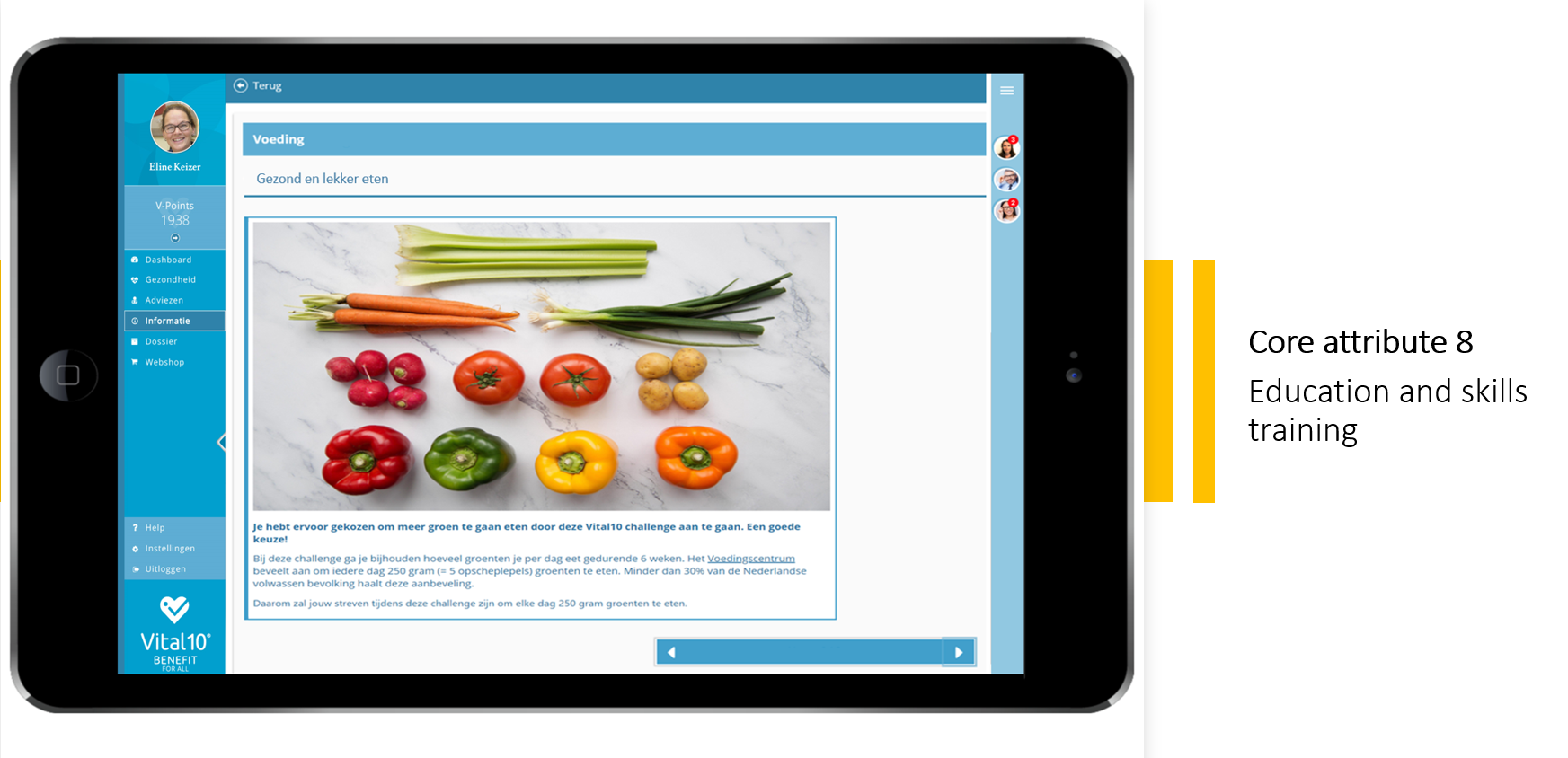


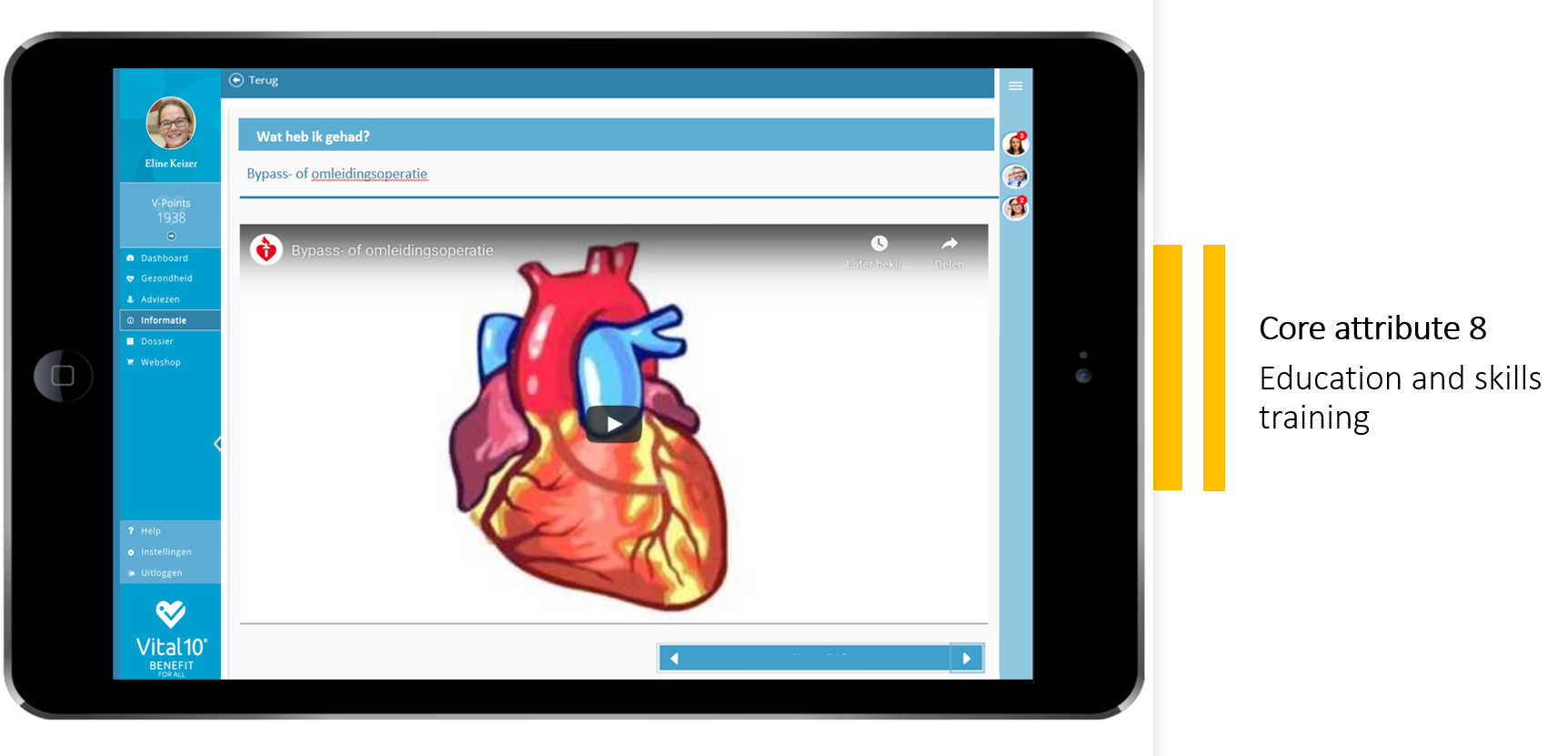


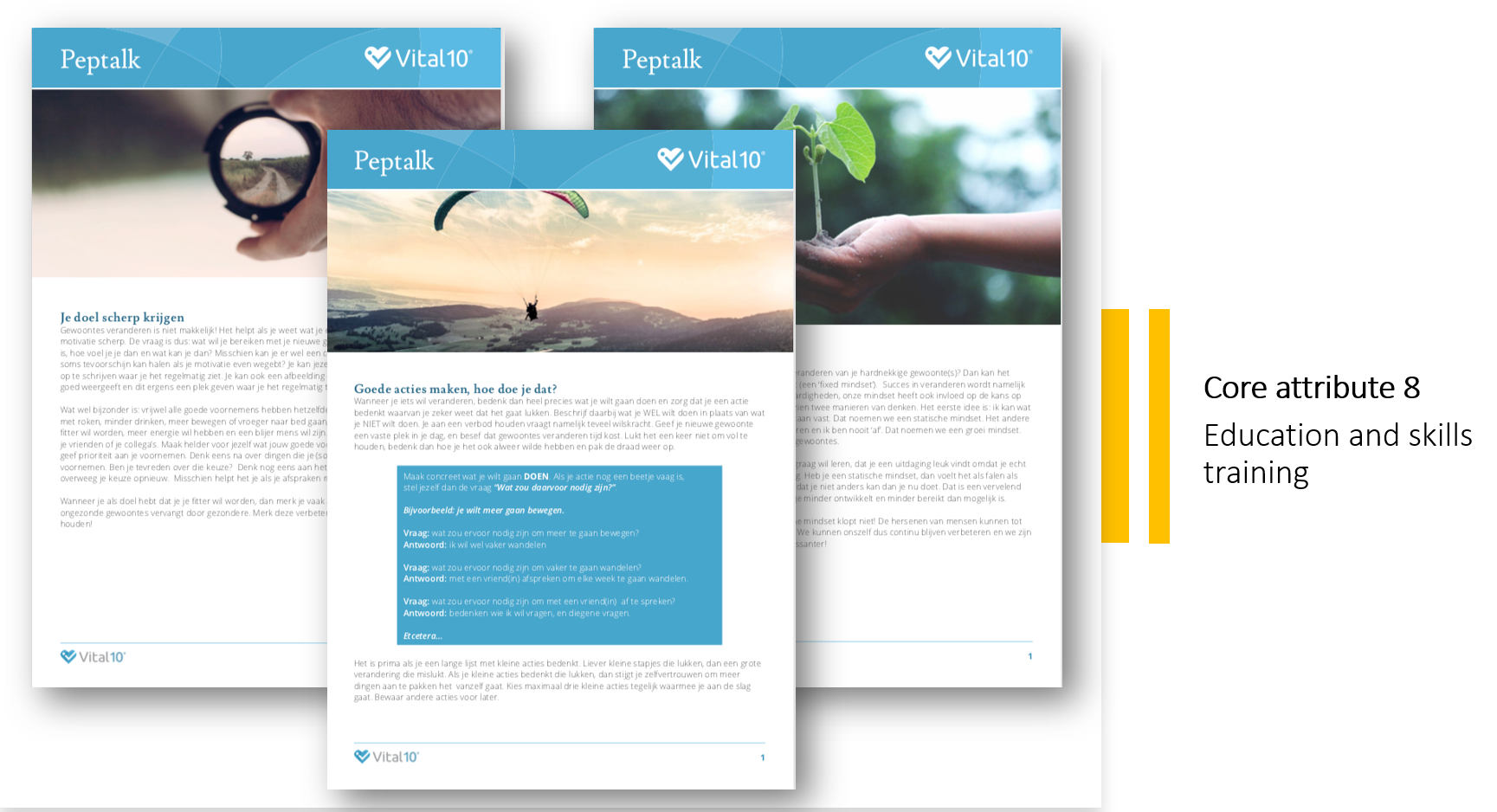


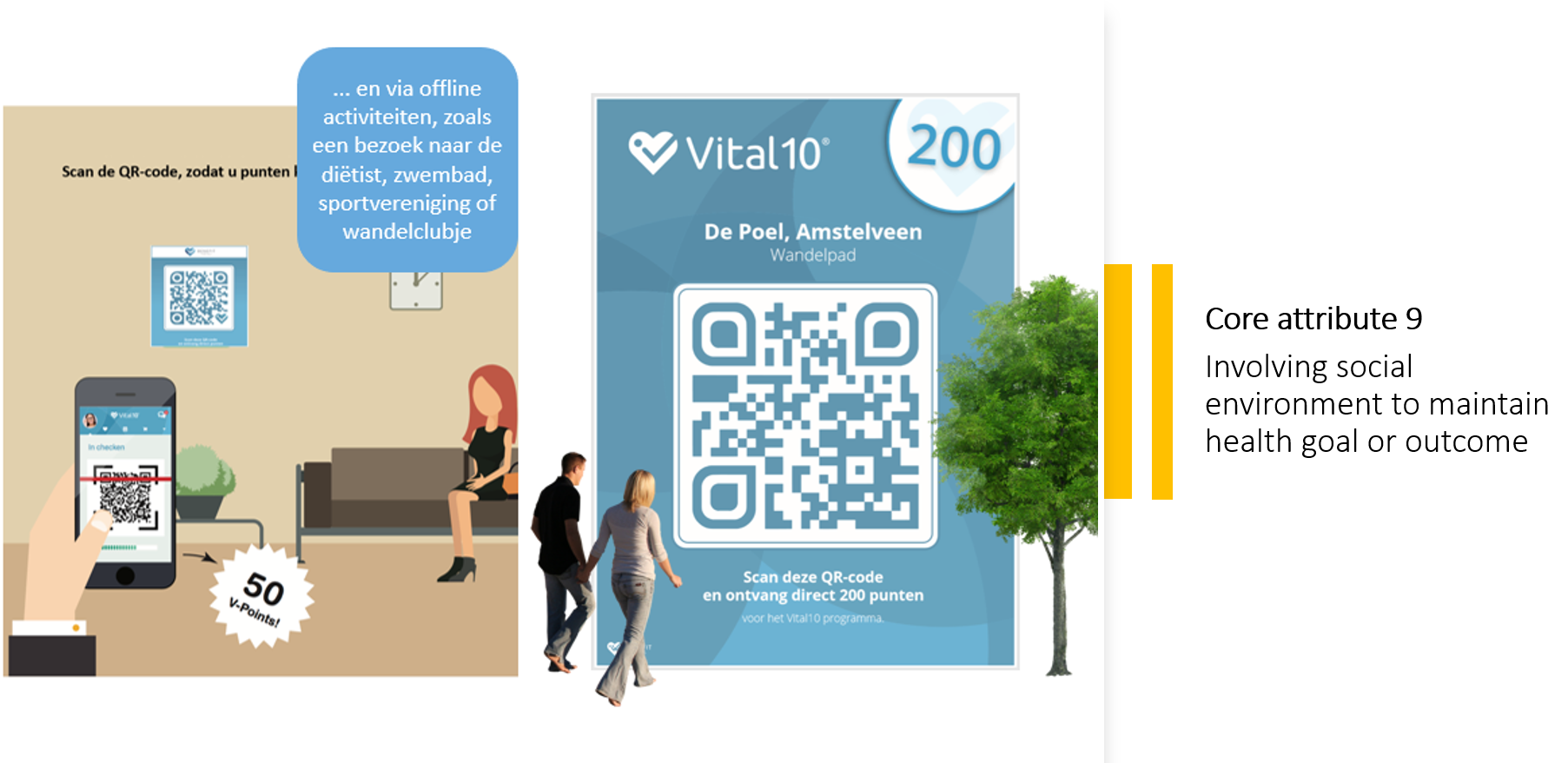


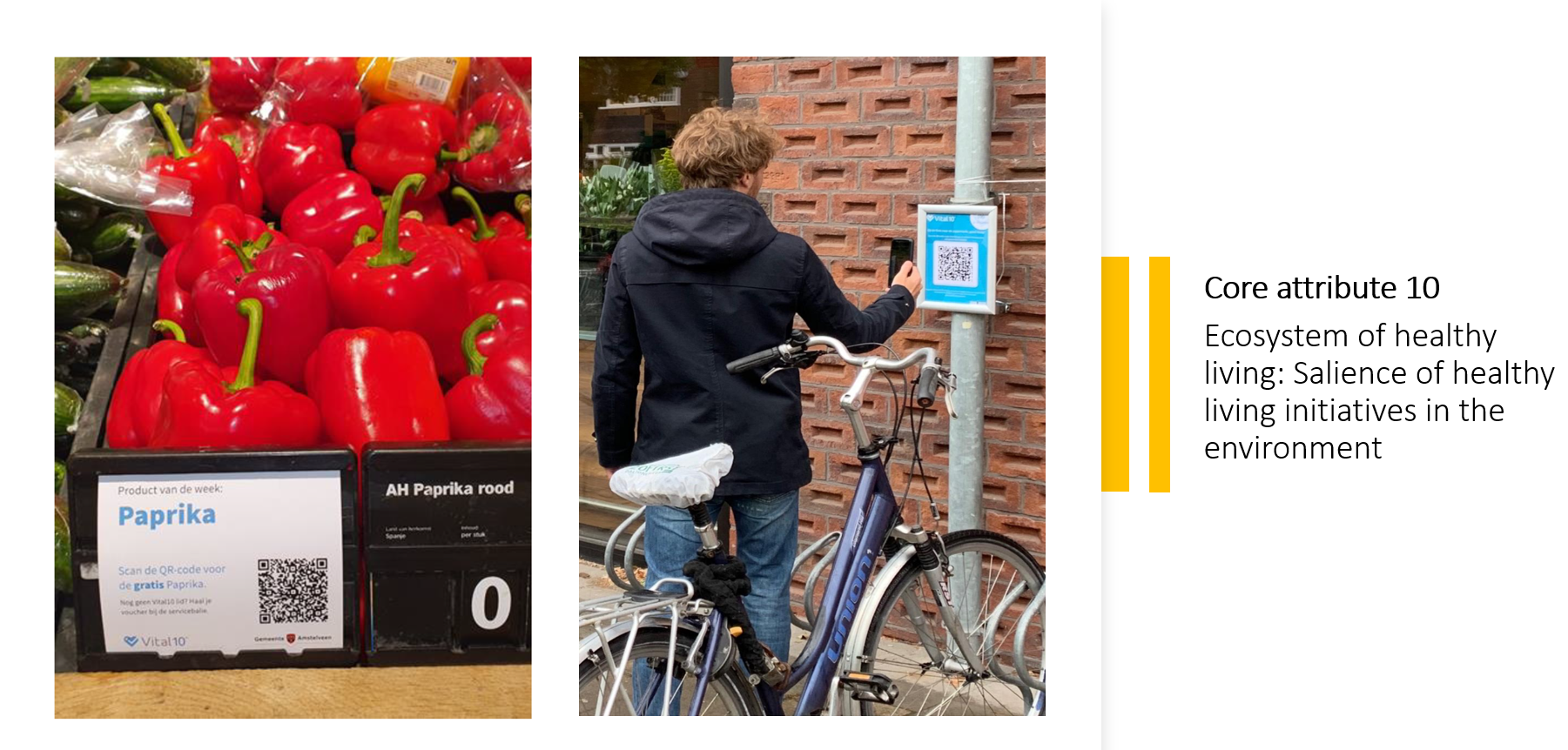


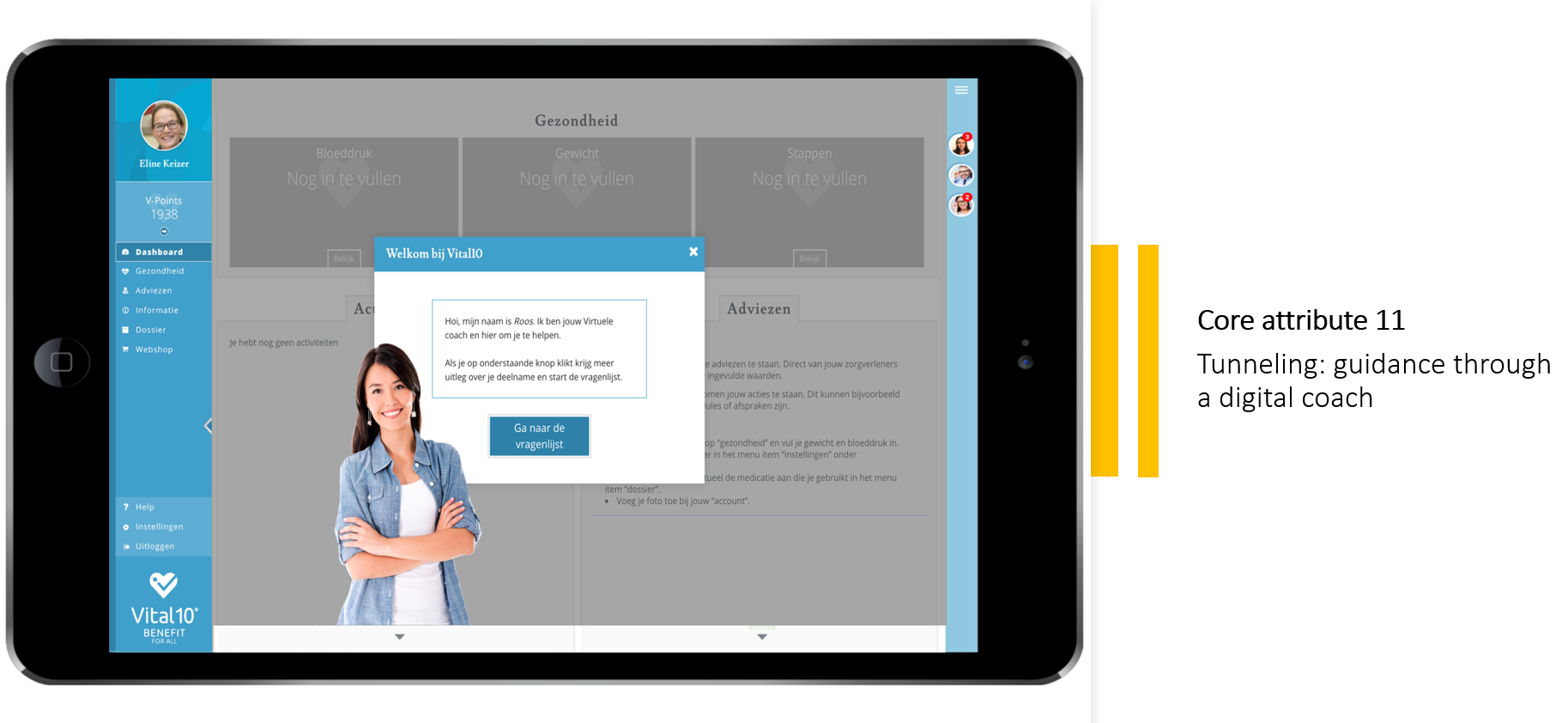


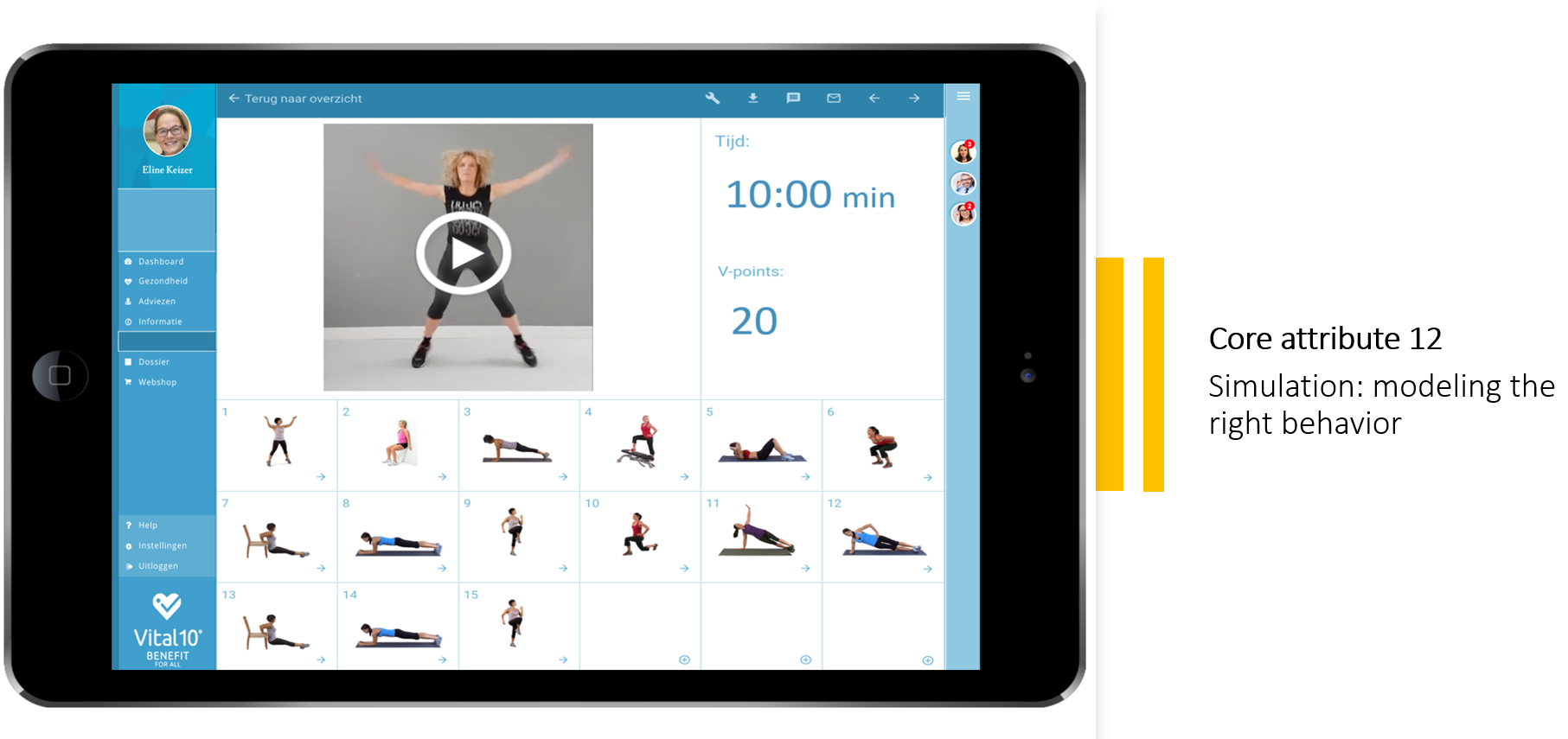


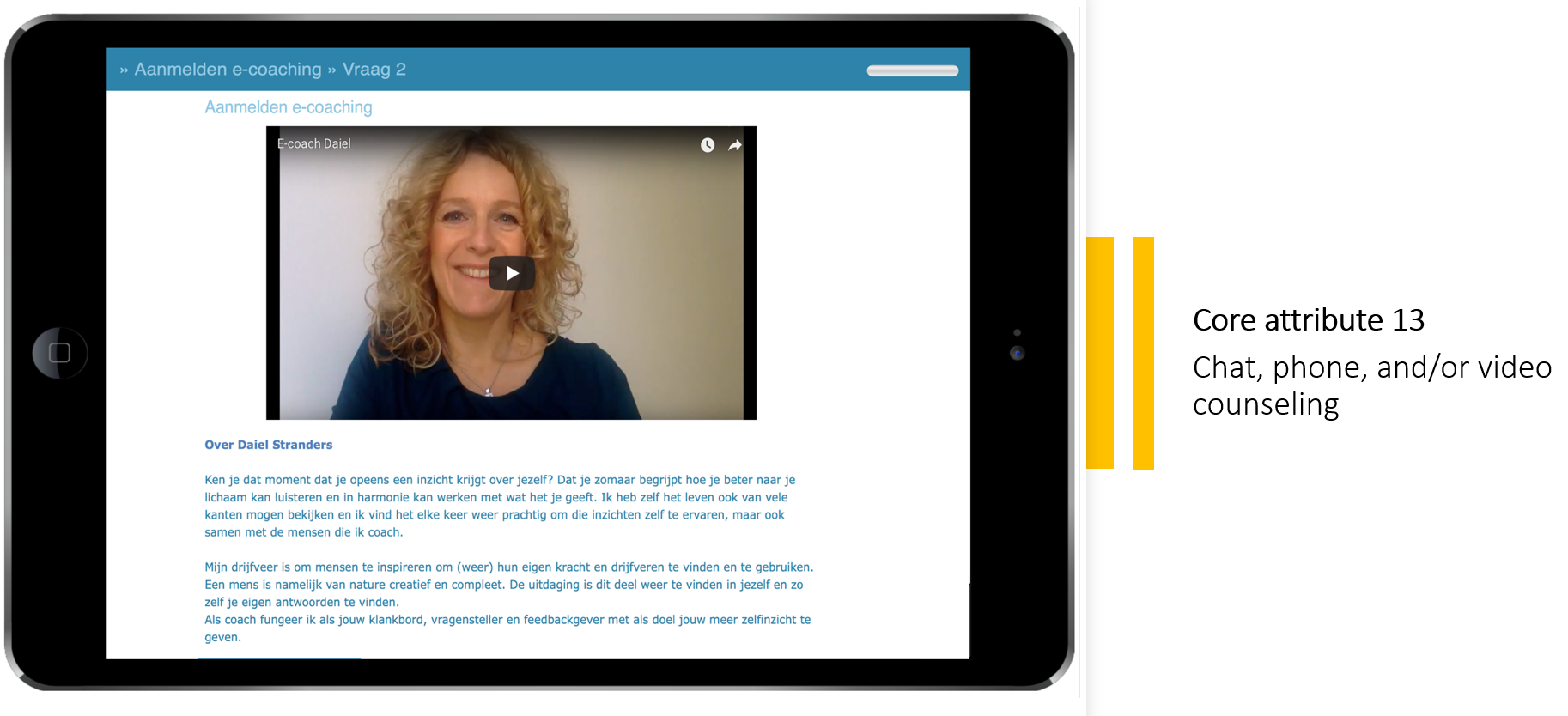


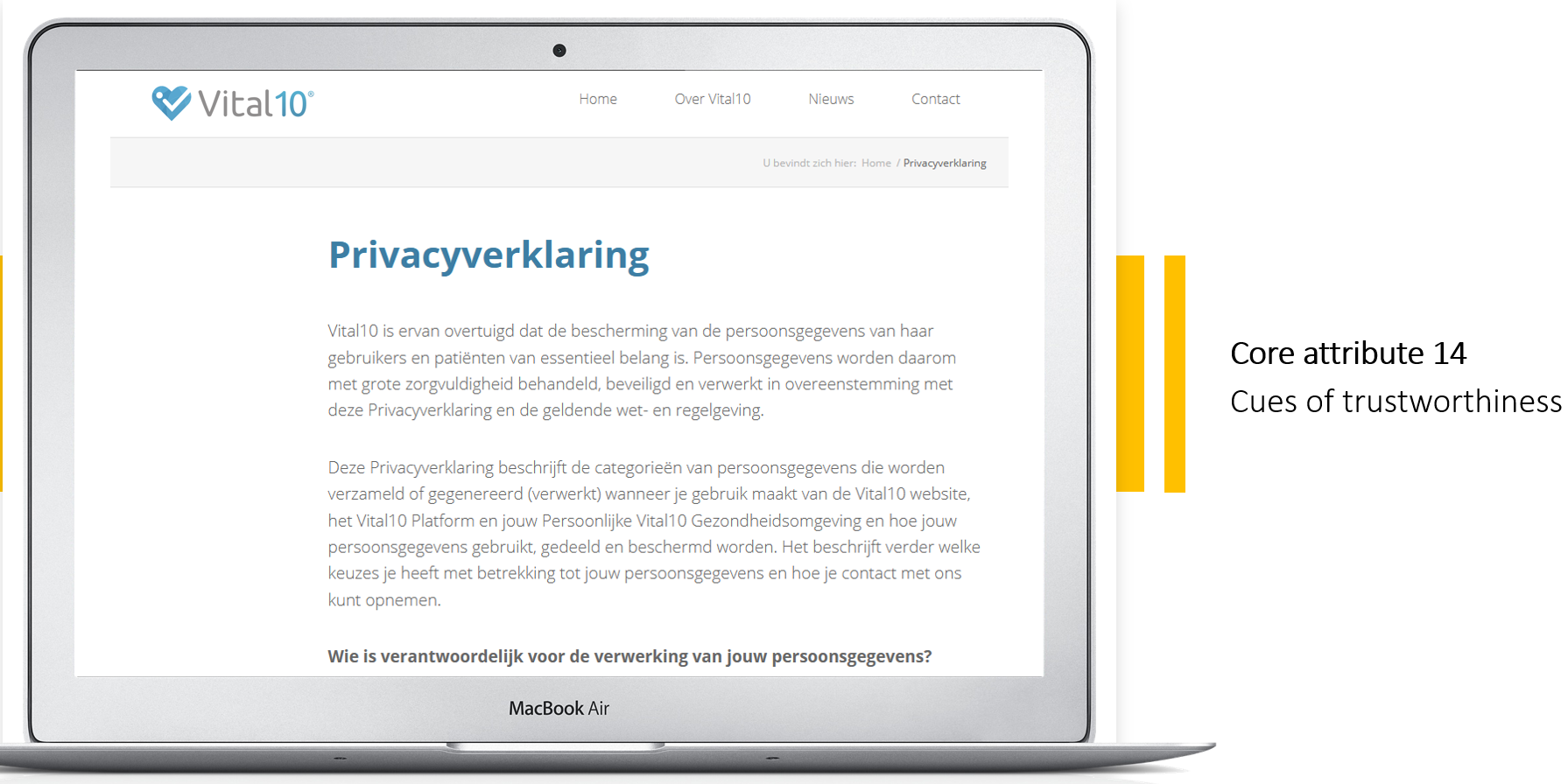

Supplement: Multimedia Appendix 1 [file cardio_v7i1e43781_app1.docx]
