# Supplementary material for: Implementation and User Evaluation of an eHealth Technology Platform Supporting Patients With Cardiovascular Disease in Managing Their Health After a Cardiac Event: Mixed Methods Study
Source: JMIR Cardio. 2023 Mar 24;7:e43781. doi: 10.2196/43781 (PMC10131764; doi:10.2196/43781)
Supplement: Multimedia Appendix 2 [file cardio_v7i1e43781_app2.docx]

## **2. Usability and interview protocol with scenarios**

Voorbereiding – voorafgaand aan gesprek

| Tijd | Taak | Doel |
| --- | --- | --- |
| Voorbereiding | 1) Participant ontvangt 2-3 dagen voorafgaand aan gesprek een mail met informatie.  Informatie over:   - De participant moet toegang hebben tot Microsoft Teams link. In deze mail wordt de link naar het gesprek gedeeld met informatie dat deze beschikbaar is vanaf datum/tijd afspraak. - Een link en inloggegevens naar het Vital10 platform - Contactgegevens van de onderzoeker (Britt) zodat de participant deze kan benaderen bij vragen voorafgaand aan gesprek. - Voeg PIF nogmaals toe - Stuur V-Cheqs naar het account!!!   *Zie voorbeeldmail op P schijf* | Het voorbereiden van de participant om deel uit te kunnen maken van de usability test. Bevat link naar teams gesprek en een link + inloggegevens naar het portaal. |

Usability test – opening gesprek

| Tijd | Taak | Doel |
| --- | --- | --- |
| *Voorbereiding aanvang gesprek* | Zorg dat je op het g-mail account bent ingelogd zodat je dadelijk de beveiligingscode tijdens het inloggen kan doorgeven. |  |
| *1 minuut* | 1) Stel je voor aan de participant en vraag de participant zich voor te stellen. Als de participant zich nog weinig openstelt, maak een leuke opmerking of stel een simpele vraag om de persoon te ontdooien, bijv.:  - Heeft u weleens eerder meegewerkt aan een gebruikerstest?  - Heeft u ervaring met beeldbellen? | Ontmoeten/kennismaken |
| *1 minuut* | 2) Vraag aan de participant of het duidelijk is aan welk onderzoek hij/zij meeneemt en geef zo nodig uitleg:  - Vertel de participant dat dit onderzoek gericht is op het testen van het gebruiksgemak van een platform gericht op het verbeteren van de leefstijl van hart- en vaatpatiënten. Ik ga u straks het platform laten zien en u een aantal opdrachten geven maar ook u zelf het platform laten ontdekken. Hierbij is het van belang dat u hardop denkt, dus uw mening met mij deelt over alles wat u ziet of denkt. Mocht u dit soms vergeten dan help ik u hieraan te herinneren. Het is hierbij niet van belang hoe u het doet op het platform, u kunt het niet goed of fout doen. Het maakt dus niks uit als u iets niet snapt of iets lastig vindt, het gaat er juist om dat u dat aangeeft zodat wij op basis daarvan het platform kunnen verbeteren.  Aanvullend op de gebruikerstest zal ik u nog wat vragen stellen, die gericht zijn op de implementatie van het platform. Hiermee kunnen we ervoor zorgen dat het platform beter bij uw wensen en binnen uw leven past. | Doel van het onderzoek en verwachte taken uitleggen |
| *1 minuut* | 3) Vraag de participant of hij/zij al eens gehoord heeft over het platform, zo niet, leg uit:  - Het doel van het platform is om u als patiënt te ondersteunen bij het aannemen en behouden van een gezonde leefstijl, en het onder controle houden van uw gezondheid en ziekte. Zoals u straks zult zien biedt het platform meerdere mogelijkheden, zoals het bijhouden van gegevens als gewicht, hartslag, bloeddruk. Daarnaast helpt het platform u bij het stellen van doelen en biedt het begeleiding bij het behalen van deze doelen, niet alleen digitaal maar ook met behulp van een coach. | Uitleggen doel platform |
| *1 minuut* | 4) Vraag of alles duidelijk is voor de participant of dat hij/zij nog vragen heeft. Zo ja, beantwoord deze naar behoefte. | Beantwoorden van vragen tot zover |
| *2 minuten* | 5) Vertel de participant dat wij voor dit onderzoek mondelinge toestemming nodig hebben voor deelname. Leg uit dat we dit gesprek opnemen en de participant vragen het scherm te delen, zodat we kunnen zien hoe de persoon deze stappen doorloopt. Dit betekent dat zowel spraak, als video opgenomen zal worden, en de participant dus ook zichtbaar zal zijn op de opname. Leg uit dat dit enkel zichtbaar is voor ons en dat wanneer uitgeschreven, alle data anoniem geanalyseerd gaat worden. Vraag na afloop of alles helder is voor de participant en of hij/zij hiermee instemt. | Uitleggen over dat we toestemming nodig hebben voor deelname/opname gesprek |
| *2 minuten* | 6) Vraag de participant de PIF uit de mail te openen. Loop het informed consent stap voor stap (oplezen) door met de participant en vraag de persoon of hij/zij toestemming geeft voor deelname. Leg uit dat we de participant na het starten van de opname dit nogmaals vragen te bevestigen, zodat we het mondeling vastgesteld hebben.  Wanneer alle stappen doorlopen zijn, vraag nogmaals aan de participant of alles duidelijk is en of de participant klaar is om te beginnen. | Toelichten van voorwaarden waar participant mee akkoord gaat bij deelname. |
| *2 minuten* | 7) Vraag de participant of hij/zij ermee akkoord gaat dat je nu de opname start, en indien ja, start de opname.  Vraag de patiënt zelf te benoemen akkoord te zijn met alle in de informed consent genoemde punten, en te bevestigen dat de participant vrijwillig instemt met deelname. | Het mondeling verkrijgen van informed consent op opname.  Let op: voor de zekerheid, neem een extra voice opname met mobiel/laptop. |
|  | 8) Vraag aan de participant of hij/zij zijn scherm kan delen. Leg uit hoe dit moet. | Let op: mocht dit niet lukken, deel dan zelf je scherm en vraag aan de participant jou door het platform heen te sturen. Vervolg verder de stappen hieronder. |

Start daadwerkelijke usability test

| Taak | Taak | Doel |
| --- | --- | --- |
|  | Extra hulpzinnen om mensen pratende te houden | - Kunt u uitleggen waarom u dit doet?  - Kunt u aangeven hoe u hierover denkt?  - Wat vindt u van deze mogelijkheid/hiervan?  - Wat vindt u van deze optie?  - Kunt u blijven vertellen wat u doet en denkt? |
| **Inloggen** | **Vraag de participant via de link uit de mail, het portaal te openen.**  *Laat de participant zijn inloggegevens (die hij/zij per mail van ons gekregen heeft) in te vullen. Kijk zelf in het g-mail account naar de binnenkomende beveiligingscode en geef deze door aan de participant.* |  |
|  | Na het inloggen komt de participant op het dashboard: **vraag om de eerste indruk.** |  |
| Invullen V-cheq | **Zoals u ziet, staat er een V-CHEQ klaar. Zou u hierop willen klikken?**  (“hoe meet ik mijn bloeddruk en geef ik de gemeten waarde door”)  *Laat zelfstandig de vragenlijst doorlopen.* Binnen de V-Cheq moet de participant de bloeddruk en hartslag invullen. *Vertel de participant dat hij/zij dit in dit geval niet hoeft te weten en getallen mag schatten*. *Benadruk dat het vooral om het onderzoeken naar gebruiksgemak gaat en het niet uitmaakt wat voor gegevens de participant invult.*  *Na het afronden en versturen van de V-cheq, laat de participant via de knop die verschijnt weer terugkeren naar het dashboard.* | Geef uitleg over de pagina en wat de respondent met deze pagina kan bereiken. Help indien nodig met invullen van bepaalde gegevens en laat de respondent vertellen wat hij/zij ziet, vindt of denkt.  Mocht de participant langer dan 1 minuut bij een onderdeel blijven haken, help de participant dan bij het invullen van desbetreffend onderdeel. |
|  | **Vraag de participant wat er nu veranderd is op het dashboard.** Als de participant geen verandering ziet, verwijs naar het blok rechts onderin, daar verschijnt nu een advies op basis van ingevulde gegevens.  **Als de participant het niet zelf ontdekt**, **vraag de participant op het advies te klikken**. *De participant mag de pagina zelf bekijken. Het is niet nodig om de gehele tekst te lezen. Als de participant vraagt of hij/zij de tekst moet lezen mag je aangeven dat het niet nodig is, mocht de participant het uit zichzelf gaan lezen, onderbreek de participant dan niet en geef de participant de tijd.*  **Vraag wat de participant ervan vindt dat er advies wordt gegeven** |  |
| +- 3 minuten | **Laat de participant weer naar het dashboard gaan. Vertel dat er een nieuwe V-Cheq klaarstaat**. *Als de participant er niet uit zichzelf op klikt, vraag de participant erop te klikken. (“dagelijks meten van de bloeddruk gedurende 7 dagen”)*  *Het betreft een korte advies pagina (1 pagina). Laat de participant vervolgens weer naar het dashboard terugkeren via de knop die verschijnt.* | Geef uitleg over de pagina en wat de respondent met deze pagina kan bereiken. Help indien nodig met invullen van bepaalde gegevens en laat de respondent vertellen wat hij/zij ziet, vindt of denkt. |
|  | Waarschijnlijk heeft de participant in het begin al aangegeven dat het hem/haar was opgevallen dat er één rode/oranje/groene tegels (gewicht) op het dashboard staat, zo niet, wijs de participant erop. **Vraag de participant wat hij/zij ervan vindt**.  Laat de participant op de tegel gewicht klikken en de nieuw geopende pagina zelf ontdekken. **Vraag de participant wat hij/zij ziet en wat hij/zij ervan vindt.**  Laat de participant zelf het gewicht invullen. *Er zal een feedback scherm verschijnen*. Laat de participant dit lezen en **vraag wat hij/zij van deze optie vindt**.  Laat de participant weer terugkeren naar het dashboard. |  |
| +- 2 minuten | Laat respondent de doelen pagina openen [dit kan niet – wel a.d.h.v. v-cheq]. Laat de participant zelf het doel invullen. [Welk doel 30 min per dag bewegen] |  |
| +- 5 minuten | Nu het doel is ingevuld, laat je de patiënt (wederom) naar de V-CHEQ pagina gaan. Hier kan de participant aangeven waar de focus ligt (van doel naar challenge). Laat de participant zelf de waarden invullen. Aan het eind kan de participant aangeven of hij/zij wil dat de coach contact met hem/haar opneemt. | Geef uitleg over de pagina en wat de respondent met deze pagina kan bereiken. Help indien nodig met invullen van bepaalde gegevens en laat de respondent vertellen wat hij/zij ziet, vindt of denkt. |
| +- 2 minuten | Laat de participant vervolgens weer naar de doelen pagina gaan. Hier is nu een overzicht van het doel van de patiënt en hoe hij/zij hier de aankomende periode naar toe gaat werken. | Geef uitleg over de pagina en wat de respondent met deze pagina kan bereiken. Help indien nodig met invullen van bepaalde gegevens en laat de respondent vertellen wat hij/zij ziet, vindt of denkt. |
| +- 2 minuten | Laat respondent de advies pagina openen. [Er moet een advies klaar staan: welk?] Laat zelf kiezen welke hij/zij wil openen en vraag wat de respondent ervan vindt. |  |
| +- 2 minuten | Laat respondent de dossier pagina openen. | Op deze pagina is er nu nog niks te zien, maar leg aan de respondent uit wat hier gezien kan worden en vraag om zijn/haar mening. |
|  | Contact/chat 🡪 je hebt extra ondersteuning nodig, het stoppen met roken lukt niet goed. Hoe kun je in contact komen met je coach? [Chat]. Je hebt een berichtje gestuurd, en je coach heeft gereageerd. [Berichten in chat klaarzetten: verwijzen naar hulpbron] |  |
| +-2 minuten | Laat respondent de hulpbronnen openen en zelf een module kiezen [moeten hulpbronnen staan] | Geef uitleg over de pagina en wat de respondent met deze pagina kan bereiken. Help indien nodig met invullen van bepaalde gegevens en laat de respondent vertellen wat hij/zij ziet, vindt of denkt. |
| +- 2 minuten | Laat respondent de webshop pagina openen | Geef uitleg over de pagina en wat de respondent met deze pagina kan bereiken. Help indien nodig met invullen van bepaalde gegevens en laat de respondent vertellen wat hij/zij ziet, vindt of denkt. |
| +- 5 minuten | Rond de usability test af. |  |

Het interview

1. **Socio-demografische gegevens**

| **A)** | **Kunt u mij kort iets vertellen over uzelf?** |
| --- | --- |
|  | - *Leeftijd, geslacht, woonsituatie, gezinssituatie (getrouwd, kinderen)* |
| **B)** | **Kunt u mij wat over uw gezondheidssituatie vertellen, of de reden waarom u het revalidatietraject volgt?** |
|  | - *Wat voor impact heeft dit op uw leven/wat zijn hiervan de gevolgen voor uw leven?* - *Hoe gaat u hier mee om?* |
| **c)** | **In hoeverre bent u zelf al bezig in het krijgen of behouden van een gezonde levensstijl?** |
|  | - *Dieet, sporten, bijhouden van gezondheidsgegevens* - *Hoe vindt u het om dit te doen?* - *Wat zou u nodig hebben om dit nog meer/beter te doen?* |

**2) Het platform**

| *Dan wil ik u nu graag het platform laten zien. Bent u al bekend met dit platform of heeft u ervaring met een soortgelijk platform?*  *Dit platform is bedoeld om u te helpen een gezondere levensstijl te krijgen of te behouden. Het helpt u met het opstellen van doelen welke u met behulp van de steun van een coach gaat proberen te behalen. Binnen het platform kunt u verschillende data bijhouden, zoals uw gewicht, bloeddruk, hartslag etc. Daarnaast biedt het platform ook adviezen op verschillende vlakken, zoals over voeding of beweging. Door uw gegevens bij te houden binnen het platform en doelen te bereiken spaart u punten die u kunt omruilen in de webshop voor kortingsbonnen voor uitjes of producten.*  *Start usability test. Tijdens het doorlopen de volgende vragen stellen:* | |
| --- | --- |
| **A)** | **Wat is uw eerste indruk van het platform?** |
|  | - Waarom? |
| **B)** | **Wat betekent het voor u dat u deze vorm van begeleiding aangeboden krijgt?** |
| **C)** | **Waarom denkt u dat dit platform ontwikkeld is, waarom zou het u aangeboden worden** |
| *Het doel van het platform is u te ondersteunen bij het aannemen en behouden van een gezonde leefstijl en het onder controle houden van uw gezondheid of ziekte. Dit kunt u op dit platform doen door doelen te stellen en daarbij ondersteuning te krijgen, informatie te vinden, uw gezondheidswaarden te monitoren en contact met zorgverleners en andere betrokkenen te onderhouden.* | |
| **D)** | **Hoe wenselijk vindt u het door een platform ondersteunt te worden bij het gezond worden en blijven?** |
|  | - Wat zou u zelf graag willen, welke doelen zou u hebben met zo’n platform? - Hoe behaalt u deze? - En hoe goed ondersteunt het platform u hierin, in hoeverre u nu kunt inschatten? |
| **E)** | **Heeft u daarnaast zelf ook doelen waarbij u denkt dat het platform kan helpen om deze te behalen?** |
|  | - Welke doelen? - Waarom is dat belangrijk voor u? - Hoe zou u met behulp van dit platform deze behalen? |
| **F)** | **Nu u het platform bekeken hebt/aan het bekijken bent, wat zou de (meer)waarde van dit platform (of tijdens usability een specifiek onderdeel aanwijzen) voor u zijn?** |
|  | - Waarom heeft dit (meer) waarde voor u? - Kunt u drie dingen/functies noemen die u op dit moment aanspreken?   - Waarom? - Zijn er ook onderdelen van dit platform die wellicht geen of minder waarde voor u hebben? |
| **G)** | **Als u het platform zo bekijkt, wat mist er dan volgens u?** |
|  | - Waarom is dit missende item van belang? - Hoe zou dit item volgens u binnen het systeem kunnen worden geïntegreerd/passen? |

**3) Het (dagelijkse) gebruik**

| **A)** | **Via dit platform kunt u heel veel zelf doen of bijhouden, in hoeverre denkt u dat dit u gaat lukken?** |
| --- | --- |
|  | - Waarom wel/niet? - Als niet: wat zou u nodig hebben om het platform zelfstandig te kunnen gebruiken?   - Kennis, zorgverleners, motivatie? |
| **B)** | *Indien participant denkt niet zelfstandig gebruik te kunnen maken:*  **Zoals u ziet heeft Vital10 ook een support desk, waar u met vragen of problemen terecht kan. Zou u met behulp van dit support desk wel zelfstandig gebruik kunnen maken van dit platform?** |
| **C)** | **In hoeverre is het gebruiken van dit platform (het bijhouden van waarden, werken aan doelen, etc.) iets wat u regelmatig (bijv. dagelijks) kunt doen? Past dit in uw dagindeling?** |
|  | - Hoe? - Wat zou u daarvan vinden? - Is dit haalbaar voor u? |

**4) Implementatie**

| **A)** | **Hoe kunnen we dit platform het beste aan nieuwe gebruikers introduceren volgens u?** |
| --- | --- |
|  | - Waar en wanneer binnen het revalidatieproces zou u de meeste behoefte hebben aan dit platform? - Door wie/op welke manier zou u het liefste kennis willen maken met dit platform? |
| **B)** | **Zou u alleen gebruik maken van het platform, of moet uw partner of mantelzorger hier ook een rol in spelen?** |
|  | - Waarom? - Op welke manier? |
| **C)** | **En wat betreft de zorgverlener, welke rol verwacht u van hem/haar wanneer u gebruikt maakt van dit platform?** |
| **D)** | **Stel, u houdt uw gemeten waarden keurig bij in het platform. Op welke wijze zou u dat dat met uw zorgverlener willen delen? Of juist helemaal niet** |
|  | - Waarom? |
